# Recent advances in melittin-based nanoparticles for antitumor treatment: from mechanisms to targeted delivery strategies

**DOI:** 10.1186/s12951-023-02223-4

**Published:** 2023-11-28

**Authors:** Xiang Yu, Siyu Jia, Shi Yu, Yaohui Chen, Chengwei Zhang, Haidan Chen, Yanfeng Dai

**Affiliations:** 1 State Key Laboratory of Digital Medical Engineering, School of Biomedical Engineering, Hainan University, Haikou, China; 2 Key Laboratory of Biomedical Engineering of Hainan Province, One Health Institute, Hainan University, Haikou, China; 3 Hubei Key Laboratory of Tumor Microenvironment and Immunotherapy, China Three Gorges University, Yichang, China; 4 The First College of Clinical Medical Science, China Three Gorges University, Yichang, China

**Keywords:** Melittin, Immunomodulatory, Side effects, Tumor, Multimechanism

## Abstract

As a naturally occurring cytolytic peptide, melittin (MLT) not only exhibits a potent direct tumor cell-killing effect but also possesses various immunomodulatory functions. MLT shows minimal chances for developing resistance and has been recognized as a promising broad-spectrum antitumor drug because of this unique dual mechanism of action. However, MLT still displays obvious toxic side effects during treatment, such as nonspecific cytolytic activity, hemolytic toxicity, coagulation disorders, and allergic reactions, seriously hampering its broad clinical applications. With thorough research on antitumor mechanisms and the rapid development of nanotechnology, significant effort has been devoted to shielding against toxicity and achieving tumor-directed drug delivery to improve the therapeutic efficacy of MLT. Herein, we mainly summarize the potential antitumor mechanisms of MLT and recent progress in the targeted delivery strategies for tumor therapy, such as passive targeting, active targeting and stimulus-responsive targeting. Additionally, we also highlight the prospects and challenges of realizing the full potential of MLT in the field of tumor therapy. By exploring the antitumor molecular mechanisms and delivery strategies of MLT, this comprehensive review may inspire new ideas for tumor multimechanism synergistic therapy.

## Introduction

The prevalence of cancer has been increasing yearly in recent years and has emerged as a global challenge and a serious threat to human health. GLOBOCAN 2020 has shown that the number of new cancer cases reached 19.3 million worldwide, and nearly 10 million people died from cancer in 2020 [1]. According to the International Agency for Research on Cancer estimates, this number of new cancer cases will increase to more than 20 million in 2025 [2]. Although there are various applications and improvements in both drugs and treatment strategies, such as chemotherapy, surgical resection, and radiation therapy, cancer remains one of the leading causes of death worldwide due to its complexity and drug resistance.

Antimicrobial peptide (AMP) is a kind of alkalescence peptide that comprises an endogenous part of the host defense system of different organisms, including mammals, plants, insects and amphibians. Based on their diverse sequences and structures, they can kill tumor cells, bacteria, and viruses by disrupting membrane integrity or inhibiting some cellular functions [3]. Melittin (MLT), the main active ingredient derived from the venom component of the European honeybee, is a 26 amino acid amphipathic cationic peptide with a hydrophobic amino-terminal region and a hydrophilic carboxy-terminal region. As a natural AMP, MLT can indiscriminately cause transient permeabilization of many different membranes at low concentrations. With the increase of the concentration, MLT readily incorporates into and disrupts cell membranes, forming pores for ion efflux, thus leading to disorder in the structure of phospholipid bilayers and the intracellular environment. Theoretically, MLT will not cause tumor cells to become resistant to antitumor agents. In addition to a direct tumor cell killing effect, MLT also possesses multiple biological functions, including gene expression regulation and immunomodulatory effects [4, 5]. Thus, MLT is a potential anticancer candidate due to its remarkable antitumor activity and immunomodulatory effects as well as its ability to overcome tumor drug resistance [6–8].

Despite the excellent cytolytic activity and anticancer performance of MLT, the serious nonspecific cytolytic activity and hemolytic toxicity largely impede its clinical applications. With the rapid development of nanotechnology, versatile nanoplatforms and strategies have been designed for the targeted delivery of MLT to reduce toxicity and improve tumor therapeutic efficacy [9]. Although some studies on MLT-based cancer therapy have been reported, these studies have never provided a comprehensive review of the antitumor mechanisms of MLT and MLT-based nanoparticle (NP) delivery strategies. Here, we review the recent progress in antitumor mechanisms and targeted delivery strategies of MLT and look forward to future research directions based on current research advances.

## Antitumor mechanisms of MLT

**Fig. 1:**
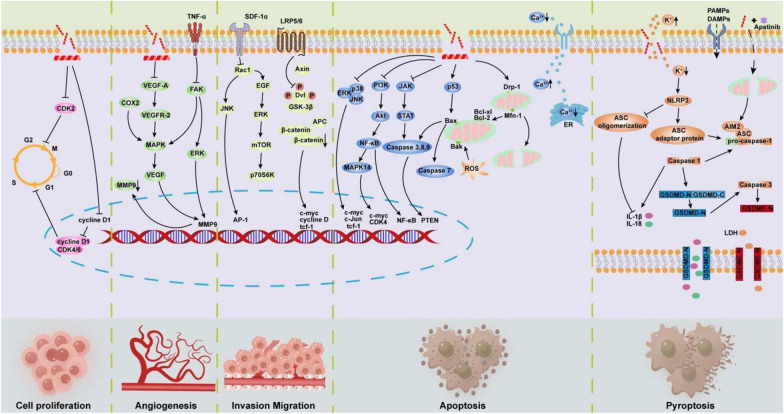
Schematic diagram summarizing the possible antitumor signal transduction pathways underlying the effects of MLT

After decades of research and exploration, MLT not only has been found to directly induce tumor cell death but also exerts antitumor activities via indirect immunomodulatory actions. In recent years, MLT has frequently been demonstrated to be an attractive antitumor drug candidate in a variety of malignant tumors via multimechanism combinations (Fig. 1).

### Inhibition of cell cycle progression

Cyclin-dependent kinase (CDK) plays a critical role in controlling various events of cell cycle regulation, including DNA repair, gene transcription, G1-S transition, and modulation of G2 progression. In the same vein, it can alter the expression of cyclins and drive aberrant proliferation in tumors [10]. More recent research has shown that MLT interacts with the CDK2 protein through 6 tight hydrogen bonds and then inhibits its activity to induce cell cycle arrest at G2/M. Meanwhile, MLT suppressed the expression of CCND1, a member of the highly conserved cyclin family, thus regulating the activity of CDK4 and CDK6 to promote the transition from G1 to S phase within the cell cycle [11, 12]. It is worth noting that the ability of MLT to induce cell cycle arrest depends on its concentration. MLT caused a slight cell cycle arrest at the G2/M phase at 0.7 µM (IC50), and the cell cycle arrest was stronger and earlier at the G0/G1 phase at 2.5 µM (IC70) [13].

### Apoptosis and necrosis

MLT dose-dependently induces apoptosis or necrosis of tumor cells. At low concentrations, MLT exhibited a strong binding affinity toward the active domain of the antiapoptotic marker Bcl-xL proteins and downregulated the expression level of Bcl2 in vitro [14, 15]. At the same time, the expression of proapoptotic markers, such as p53, Bcl-2-associated X protein (Bax), cysteinyl aspartate specific proteinase (caspase) 3, caspase 7 and the tumor suppressor phosphatase and tensin homolog (PTEN), were significantly upregulated. MLT was proven to regulate multiple cellular and molecular pathways associated with apoptosis, such as the JAK/STAT and PI3K/Akt pathways, to develop an antitumorigenic effect [16]. It was also found to induce chronic myeloid leukaemia cell death via modulation of the NF-κB/MAPK14 axis, inhibition of c-MYC and CDK4, and upregulation of JUN genes [17]. MLT not only activates caspases, the main component of the molecular mechanism of apoptosis, but is also involved in intrinsic/mitochondrial-dependent pathways [18]. Mitochondrial dysfunction has been suggested to be a contributory factor and even central to the induction of the apoptotic pathway, which leads to mitochondrial outer membrane permeabilization (MOMP). Research has suggested that the proapoptotic effects of MLT on 4T1 breast cancer cells are associated with the upregulation of mitofusin 1 (Mfn1) and dynamin-related protein 1 (Drp1) [19]. In fact, Mfn1 has been shown to facilitate apoptosis by activating the pro-apoptotic Bcl-2 family protein Bak [20]. Drp1 is involved in both mitochondrial fission and cristae remodeling and plays a dual role during apoptosis [21]. Recently, Ceremuga et al. suggested that MLT induced a potent loss in the mitochondrial membrane potential (ΔΨm) generated by proton pumps and Ca^2+^ release from the endoplasmic reticulum [22]. Although the antitumor effect of MLT can be attributed in part to the nonspecific killing of proliferating cells, some studies have suggested that MLT specifically targets cancer cells to induce cell death. Yan et al. found that MLT blocked the maturation of miR-146a-5p by selectively targeting methyltransferase-like protein 3 (METTL3) and subsequently stimulated the NUMB/NOTCH2 pathway to induce bladder cancer cell apoptosis [23]. Further investigation revealed that METTL3/miR-146a-5p/NUMB/NOTCH2 signaling was positively correlated with recurrence, metastasis, and survival in bladder cancer patients, indicating that MLT sheds new light on therapeutic targets for recurrent bladder cancer treatment.

In addition to the pro-apoptotic effects, MLT induced tumor necrosis at high concentrations. For example, 2.5 µM MLT (IC70) increased the proportion of late apoptotic and necrotic cells [13]. Significantly, a higher concentration of MLT could induce the loss of plasma membrane integrity and the consequent leakage of cellular contents. Previous research has shown that high concentrations of MLT (20 μg/mL) induce cell membrane damage in gastric and colorectal cancer cells within 1 min. Then, significant diffusion spanned across the entire bilayer over time, further causing cell membrane disruption and intracellular material expulsion from the cells, followed by complete cell necrosis occurring over a period of 15 min [24]. Furthermore, cell damage could also lead to the release of tumor necrosis factor (TNF)-α, interleukin (IL)-1β, IL-2, and interferon-γ (IFN-γ) [25–27]. Rocha et al. reported that necrosis and inflammatory infiltration were observed in bone metastasis from colorectal cancer after intrametastatic injection of a single dose of 1.5 mg/kg MLT. Ultimately, it inhibited approximately 50% of the growth of bone metastasis in colorectal cancer, providing insight into the ability of MLT to induce necrosis and its effect on tumor growth control [28].

### Inhibition of malignant biological behavior

Tumor cells present complex behaviors in their interactions with other cells, including proliferation, migration, invasion, angiogenesis, and uncertain malignant potential. The evaluation and interference of malignant biological behaviors could provide insights into tumor heterogeneity and the tumor microenvironment (TME) while providing clinically relevant metrics for tumor classification and relevant treatments [29]. MLT has been shown to provide a potential antiangiogenic effect to suppress vascular endothelial growth factor (VEGF)-induced tumor growth by blocking vascular endothelial growth factor receptor-2 (VEGFR-2) and the cyclooxygenase-2 (COX-2)-mediated mitogen-activated protein kinase (MAPK) signaling pathways [30]. It also decreased hypoxia-inducible factor 1α (HIF-1α) protein synthesis by inhibiting the ERK/mTOR/p70S6K pathways. Moreover, MLT showed an antiangiogenic effect by decreasing VEGF expression [31]. It is known that the overexpression of TNF-α can upregulate the expression of matrix metalloproteinases (MMPs) 2 and 9 via FAK/ERK signaling activation, which is important for angiogenesis, invasion, and metastasis. Bakary et al. found that MLT downregulated the levels of NO and VEGF and reduced MMP2 and MMP9 activities to suppress tumor cell proliferation, angiogenesis, and invasion [32]. Ras-related C3 botulinum toxin substrate 1 (Rac1), a well-studied Rho GTPase of the Rho family, is involved in the activation of c-Jun N-terminal kinase (JNK) and JNK-dependent cell motility. Meanwhile, Rac1 also mediates numerous basic cellular processes, such as actin cytoskeleton regulation, mesenchymal-like migration, and cellular mechanosensing [33]. Previous research has shown that MLT can prevent liver cancer cell metastasis by inhibiting Rac1-induced cell migration [34]. It has also been revealed that MLT can inhibit the migration and invasion of epidermal growth factor (EGF)-induced MDA-MB-231 tumor cells by blocking the SDF-1α/CXCR4 and Rac1-mediated signaling pathways [35].

### Pyroptosis

As a new form of programmed cell death mechanism, pyroptosis is characterized by rapid disruption of cell swelling and plasma membrane, followed by the release of intracellular contents and proinflammatory cytokines, such as IL-1β and IL-18 [36]. Recent research has suggested that pyroptosis-induced inflammation triggers a cytokine cascade yielding the release of danger-associated molecular patterns (DAMPs) to recruit immune cells to fight a tumor [37, 38]. NOD-like receptor thermal protein domain associated protein 3 (NLRP3) inflammasome activation results in the production of active IL-1β and IL-18, as well as the occurrence of pyroptosis [39]. A previous study showed that MLT can decrease the intracellular K^+^ concentration in macrophages, induce NLRP3 inflammasome formation, and increase caspase 1 activity [40]. It is well known that the NLRP3 inflammasome requires the adaptor protein apoptosis-associated speck-like protein containing a CARD (ASC) to activate caspase 1. However, MLT resulted in a failure to form large ASC oligomeric signaling complexes, thus preventing the further execution of pyroptosis by caspase 1. Moreover, the excessive rate of cell death caused by rapid cellular lysis led to reduced NLRP3 inflammasome activation. Therefore, they indicated that rapid cell lysis driven by MLT excluded caspase 1-dependent pyroptotic cell death [40]. Nevertheless, Zhao et al. recently described that MLT in combination with apatinib increased cleaved caspase 1 and the N-terminal fragment of gasdermin D (GSDMD-N) to induce synergistic antitumor efficacy in a xenograft tumor model [41]. Furthermore, it was found that MLT could cause the release of mitochondrial DNA into the cytoplasm and activate another Nod-like receptor absent in melanoma 2 (AIM2), thereby promoting the recruitment of pro–caspase 1. Ultimately, pyroptosis was triggered through a two-way positive feedback interaction between the caspase 1-GSDMD and caspase 3-GSDME axes. However, this process is unrelated to the generation of reactive oxygen species (ROS) or NLRP3 activation. These data not only provide insight into understanding the antitumor mechanism of MLT but also offer a new direction for the antitumor effects mediated by pyroptosis.

### Immunogenic cell death (ICD)

**Fig. 2:**
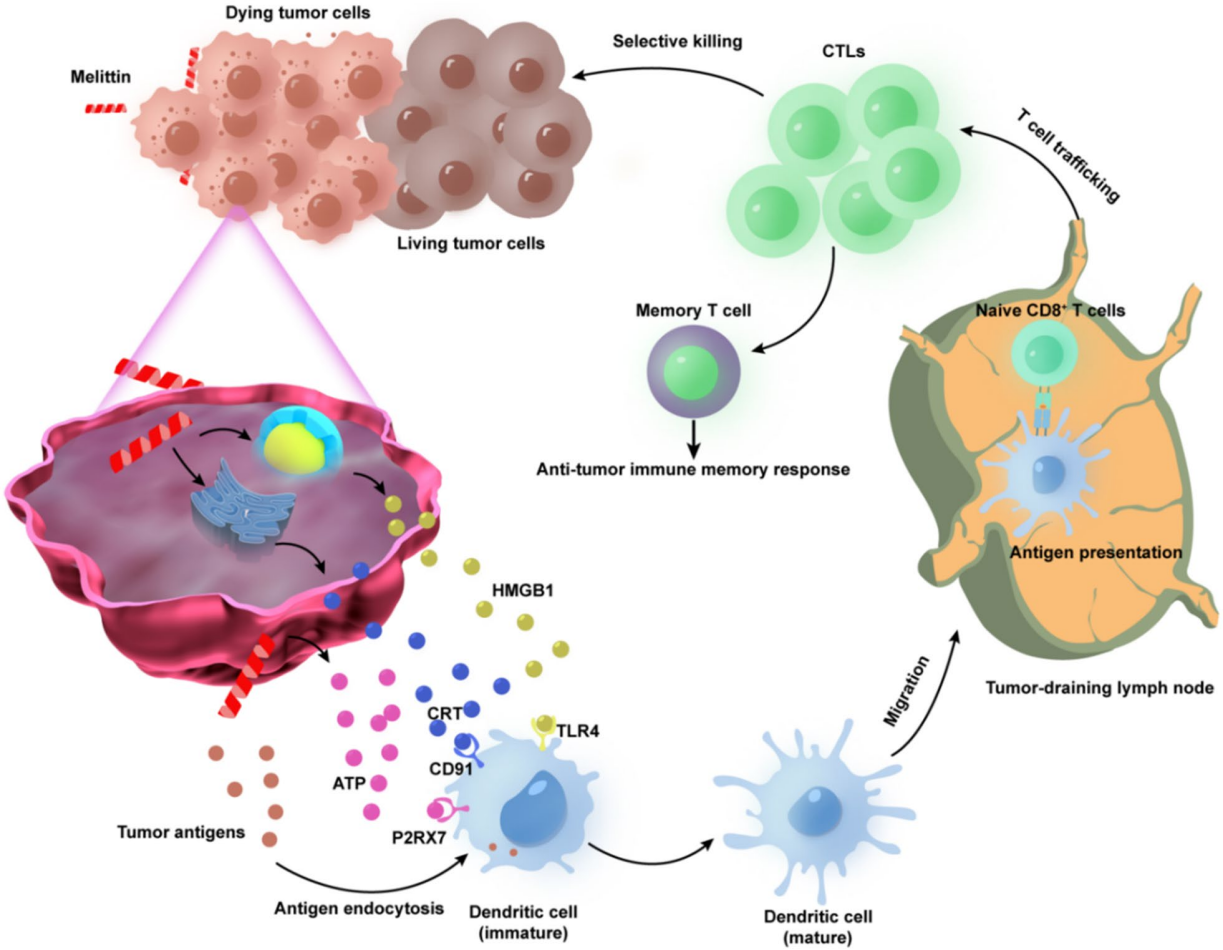
Schematic diagram of immunogenic cell death (ICD) induced by MLT

Cancer immunotherapy faces some serious challenges because of limited lymphocytic infiltration and immunosuppression. Tumor cells undergoing ICD provoke immunostimulatory effects owing to the exposure or release of DAMPs, such as heat shock proteins (HSPs), calreticulin (CRT), the high-mobility group box 1 (HMGB1) protein, and adenosine triphosphate (ATP). Immune responses require direct recognition of these DAMPs through pattern recognition receptors (PRRs) on dendritic cells (DCs), which facilitates the maturation of DCs and increases T cell priming. ICD is therefore generally considered one of the necessary conditions for MLT-based cancer immunotherapy (Fig. 2). Lv et al. constructed D-MLT micelles (DMMs) by substituting l-amino acids with d-amino acids without compromising the bioactivity of the peptide. The polymer encapsulation of D-melittin permitted higher peptide dosing (5 mg peptide/kg) and exhibited significant antitumor effects and extended survival [42]. In addition, DMM was verified to promote CRT surface expression, ATP secretion, and HMGB1 extracellular release. Innate immune responses to tumor cells are induced by exposing CRT on the cell surface, leading to antigen presentation and productive adaptive antitumor responses [43]. At the later stage of ICD, HMGB1 released from necrotic cells facilitates Toll-like receptor 4 (TLR4)-mediated antigen processing in antigen presenting cells (APCs), thereby triggering antigen-specific antitumor T cell responses [44, 45]. It is well known that photodynamic therapy (PDT) is usually insufficient to trigger effective ICD to promote strong host adaptive immune activation. MLT can regulate cell membrane permeability and promote the release of intracellular contents, including tumor antigens and DAMPs. Thus, MLT might effectively contribute to the ICD triggered by PDT to achieve a stronger antitumor immune response to inhibit primary tumor growth and tumor metastasis. Liu et al. developed a multifunctional platform, Ce6/MLT@SAB, to facilitate the penetration of NPs and accumulation in target cells by MLT-induced transmembrane pores [46]. Furthermore, they could generate an effective ICD response and activate dendritic cells following 660 nm light phototreatment to increase antigen presentation and provoke systemic antitumor immunity. A single injection of Ce6/MLT@SAB combined with phototreatment eradicated one-third of subcutaneous tumors in treated mice.

### Ferroptosis

Ferroptosis is a newly identified form of programmed cell death that is caused by glutathione (GSH) depletion and iron-dependent lipid peroxidation [47]. Extensive preclinical evidence suggests that the induction of tumor cell ferroptosis might be an effective therapeutic strategy [48]. TMEM16/ANO proteins were identified as a family of proteins that can operate Ca^2+^-activated Cl^−^ channels and phospholipid scramblases. As a potent activator of PLA2, MLT can activate Cl^−^ currents both by TMEM16A/ANO1 and TMEM16F/ANO6 [49]. The reaction of hydrogen peroxide (H_2_O_2_) with Cl^−^ can produce hypochlorous acid (HOCl), which plays a pivotal role in ferroptosis processes [50]. It was found that activation of TMEM16 by lipid peroxidation may be closely related to inflammation, proliferation, hypoxia/reperfusion, ion secretion and ferroptosis. It has also been shown that ANO1 and ANO6 can be activated during ferroptotic cell death [51, 52]. Therefore, ferroptosis has a potential role in cell death induced by MLT. Recent studies also confirmed that MLT induced ROS bursts and disrupted the GSH-glutathione peroxidase 4 (GPX4) antioxidant system to increase lipid peroxide accumulation. MLT also upregulated intracellular Fe^2+^ levels and activated the ER stress-C/EBP homologous protein (CHOP) apoptotic signal, indicating that ferroptosis was involved in the A549 cell death induced by MLT [53].

### Immunomodulatory functions

As a cationic host defense peptide, MLT exerts a variety of profound immunomodulatory effects to inhibit the initiation and development of tumors. Tumor-associated macrophages (TAMs), which are the most abundant immune-related cells in the TME, are considered to have an M2-like phenotype and participate in tumor development by mediating angiogenesis, metastasis, and immune escape [54]. It was found that MLT slightly decreased IL-10 production and inhibited M2 macrophage differentiation [12]. Although some studies have deemed it possible that M2 macrophages are reprogrammed to the M1 type, the specific mechanism remains to be further elucidated. In addition, Han et al. reported that MLT-d(KLAKLAK)_2_ (MLT-KLA) can bind preferentially to M2-like macrophages to induce more apoptosis of M2-like TAMs and inhibit the proliferation and migration of M2 macrophages, resulting in a decrease in melanoma tumor growth [55]. In addition to macrophages, professional and nonprofessional antigen-presenting cells are also involved in the immune activation effects of MLT. Previously, we developed an ultrasmall (10–20 nm) MLT-lipid nanoparticle (named α-melittin-NP) that can selectively activate liver sinusoidal endothelial cells (LSECs) and reverse the immunosuppressive microenvironment in the liver in a concentration-dependent manner. After α-melittin-NP treatment, the liver immune microenvironment had significant changes in multiple cytokines and chemokines, such as IL-18, IL-1α, chemokine (C-X-C motif) ligand 9 (CXCL9), CXCL10, chemokine (C–C motif) ligand 3 (CCL3), CCL4, CCL5, and CXCL13, thereby generating protective T-cell immunity through coordination with NK cells to inhibit liver metastasis [56]. Furthermore, we demonstrated that α-melittin-NPs also induced the maturation of macrophages and DCs in lymph nodes (LNs) and caused dramatic changes in the cytokine/chemokine milieu in the tumor, thus successfully eliciting systemic humoral and cellular immune responses [57].

## Toxic side effects of MLT

**Fig. 3:**
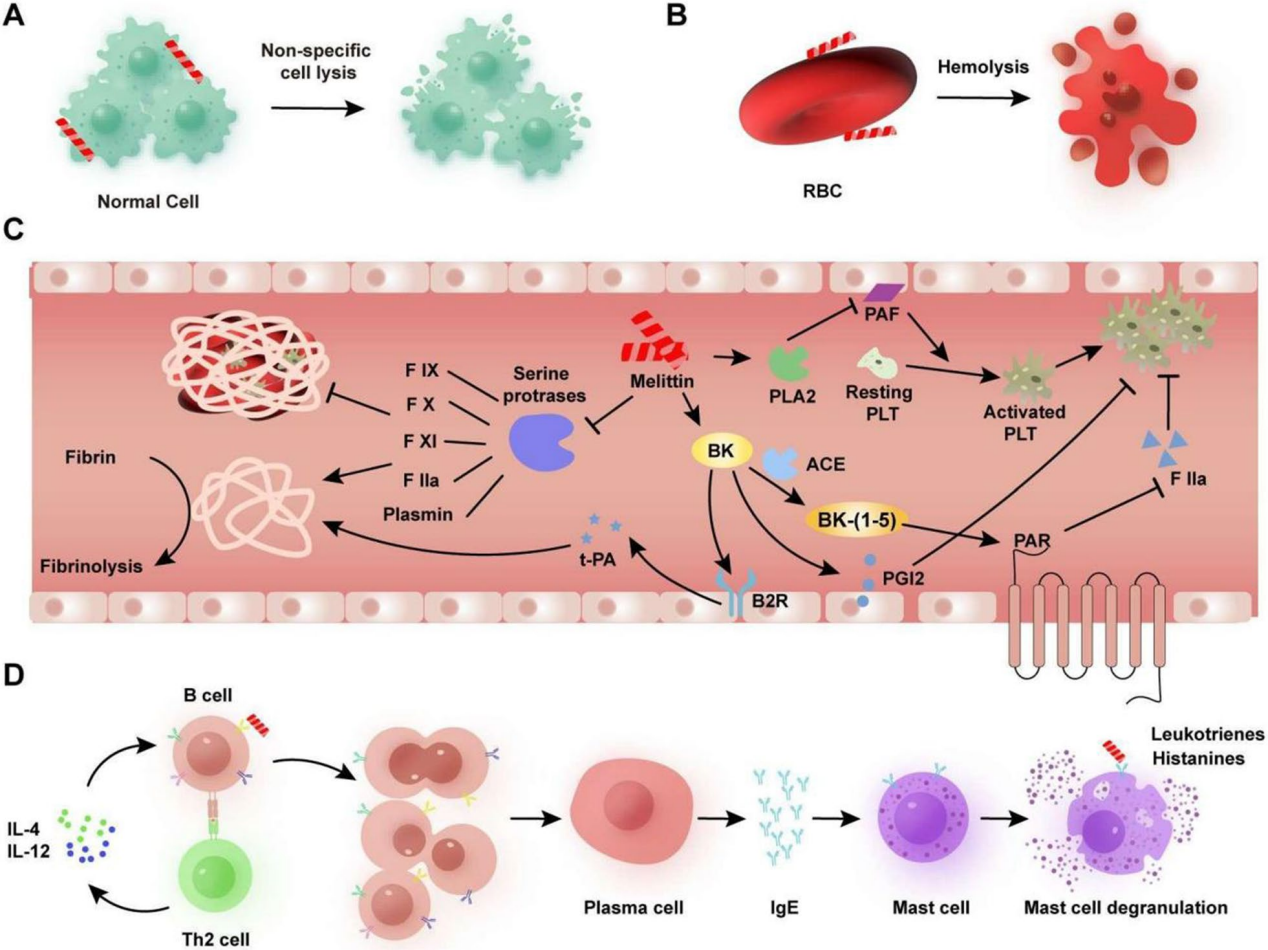
Direct exposure to MLT in vivo could lead to toxic side effects such as **A** nonspecific cell lysis, **B** hemolysis, **C** coagulation disorders, and **D** allergic reactions

Although MLT can inhibit tumor progression and metastasis through various mechanisms, the narrow range of safe doses of MLT hinders its applications in vivo. MLT often causes detrimental side effects at therapeutically effective concentrations, including nonspecific cell lysis, hemolysis, coagulation disorders, allergic reactions and so on (Fig. 3). In addition, low concentrations of MLT are genotoxic because of its DNA damaging effects [58]. This section summarizes the safety issues of MLT and the potential mechanism underlying its toxic side effects.

### Nonspecific cell lysis

MLT acts mainly by its natural detergent-like effect on the plasma membrane to lower the surface tension of water at the level of the plasma membrane [59]. The increased membrane permeability occurs simultaneously, causing rapid cell lysis with the initiation of cell death. More specifically, MLT can transiently adsorb to the surface of negatively charged biological membranes and insert into the hydrophobic core of the phospholipid bilayer due to positive charge entrainment [60]. Then, transmembrane pores will be produced through “toroidal pores”, “barrel-stave” or “carpet” mechanisms and collapse the phospholipid bilayer to promote cell lysis as well as transient cell membrane permeabilization [61–63]. Therefore, as a nonselective cytolytic peptide, MLT can disrupt almost all prokaryotic and eukaryotic cells by altering cellular membranes physically and chemically. Maher et al. showed that MLT exhibited significant toxicity in two distinct intestinal epithelial cell lines (HT29 and Caco-2) in a concentration- and time-dependent manners [64]. Phase contrast microscopy showed that signs of human umbilical vein endothelial cell (HUVEC) morphological changes were already detected after 5 min of exposure to 10 μg/mL MLT. These changes gradually increased and were mainly characterized by an increasing quantity of extracellular vesicles attached to the cell surface, granulated cell morphology, and shrinkage of cells [65]. Significantly, MLT also asserts toxicity in vivo. In early-stage research, MLT (4 μg/g) caused delta lesions, hypercontraction of myofibrils, and even necrosis of skeletal muscle cells within 30 min after i.m. injection [66]. High doses of MLT (≥ 30 μg/dose) provoked inflammation and local pain and even caused death with hypothermia, ataxia, hepatotoxic effects, and loss of weight [67]. The nonselective cytolytic activity and damage induced by MLT over a range of concentrations suggest that it is not suitable for the treatment of either topical or systemic administration in the clinic.”

### Hemolysis

MLT needs to be used via parenteral routes, including intravenous injection, intraperitoneal injection, and subcutaneous implantation because of the limitations of peptide-based medicines, such as low oral bioavailability and short plasma half-life. Nevertheless, as a small polypeptide with a molecular weight of only 2846 Da, MLT preferentially entered the blood circulation and was filtered through the glomerulus and rapidly metabolized after subcutaneous injection. Therefore, to adopt MLT as a biopharmaceutical for solid tumor targeting, its behavior in the bloodstream is of great importance [68]. Accumulating data suggest that MLT has strong hemolytic activities in RBCs. A previous study found that MLT caused 100% hemolysis at a concentration of 8 μg/mL, as measured by the increased absorbance of RBC-released hemoglobin [69]. Since RBC membranes are mostly neutral, highly charged peptides are not anticipated to induce severe hemolytic activity. For example, the antimicrobial peptide cupiennin with a high charge (+ 8) did not exhibit significant hemolytic activity, similar to MLT [4]. In fact, amino acids, such as Trp, Lys, and Arg, play an important role in the hemolytic activity of MLT [70]. It has been reported that the Trp residue of MLT is involved in the binding of peptides to cholesterol present in biological membranes through the indole moiety [71]. In addition, prior research indicated that the heptadic leucine of MLT had a direct impact on the helical assembly in an aqueous environment, the secondary structure, and membrane permeability, thus causing potent hemolysis activity [72].

### Coagulation disorders

It is commonly known that the mechanism of coagulation involves a series of reactions, such as activation, adhesion, and aggregation of platelets, along with deposition and maturation of fibrin [73]. As the principal active component of bee venom (BV) and a powerful stimulator of PLA2, MLT has been proven to increase the blood clotting time in vitro [74]. Previous research has shown that MLT can induce the release of bradykinin (BK) in association with angiotensin-converting enzyme (ACE) dysfunction, thus leading to the inhibition of platelet aggregation, coagulation disorders and fibrinolysis [75]. In addition, MLT interferes with complement cleavage. Both mechanisms are directly or indirectly associated with coagulation and thrombolysis [76]. Serine proteases play a major role in coagulopathies and act by driving thrombotic and thrombolytic cascades [77]. When inactive serine protease enzymes and their glycoproteins are activated, the next reaction in the cascade can be catalyzed, leading to coagulation in the blood [78]. However, MLT can inhibit the activity of serine proteases and effectively perturb the blood-clotting cascade to delay clotting [79]. In this case, the clotting system is often unable to achieve the desired hemostatic effect [80]. Due to the fibrinogen decrease and a moderate delay in prothrombin and partial thromboplastin times, MLT can result in skin petechiae, wound bleeding and episodic hemorrhage (especially metrorrhagia) [75, 81].”

### Allergic reactions

BVs, a well-known trigger of the allergic response, can induce a range of reactions from mild and local symptoms such as pain, swelling, redness and itching to immediate life-threatening anaphylaxis manifested as shock, laryngospasm, and respiratory failure [82]. BVs often induce an IgE response in approximately one-third of honeybee venom-sensitive patients [83]. Among all of the components of the BV complex mixture, MLT shows weaker allergens compared to other potent allergens in BV, such as PLA2, followed by hyaluronidase and icarapin [84]. However, available studies suggest that currently available MLT potentially contains residues of strong allergens such as hyaluronidase, PLA2 and acid phosphatase [83]. MLT has been reported as a major allergen in 69 patients with allergies to *A. mellifera* venom, whose prevalence of sensitization to MLT was 53.6% [85]. Furthermore, MLT can produce persistent pain hypersensitivity when injected subcutaneously in the periphery [86]. Previous studies have indicated that the activation of peripheral P2X and P2Y receptors, transient receptor potential (TRP) vanilloid receptor 1 (TRPV1) and canonical TRP channels might be involved in the pathophysiological processing of MLT-induced hypersensitivity [87, 88]. However, the molecular mechanisms by which the innate immune system initiates allergic responses remain largely undiscovered [89].

## Targeted delivery strategies of MLT for tumor therapy

**Fig. 4:**
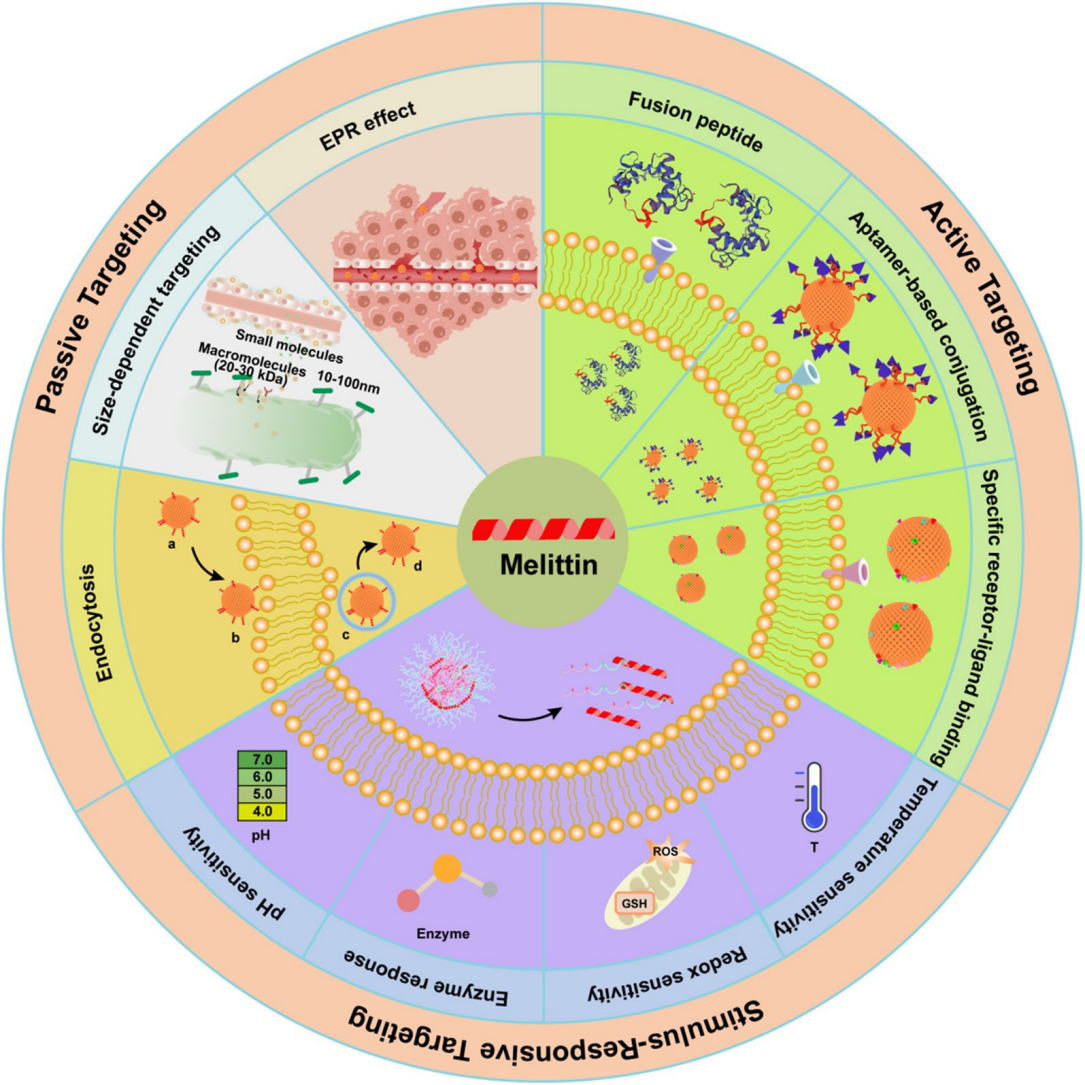
Schematic diagram of targeted delivery strategies of MLT for tumor treatment

To further improve the antitumor effect of MLT-based drugs, various strategies have been developed to minimize undesirable side effects and improve the tumor-killing effect. Some studies have attempted to alter the sequence or fine-tune the conformation of MLT to address the above issues with the goal of decreasing nonspecific hemolysis [69, 90]. However, the effects were not significant or even inevitably led to a decrease in the membrane lytic activity of MLT on tumor cells. To further improve the tumor cell-specific cytotoxicity of MLT, smart nanocarrier-based drug delivery strategies have been developed to achieve passive targeting or active targeting for the treatment of relapsed and refractory malignancies. In recent years, various classes of NPs have attracted considerable attention in the field of biomedical research due to their advantages, including appropriate pore size, ultrahigh specific surface area, ease of surface modification, and excellent biocompatibility. Additionally, based on the differences in receptors on the surface of tumor cells and normal cells or specifically responding to endogenous or exogenous stimuli at the targeted site, researchers are making elaborate efforts to develop functionalization strategies, such as active targeting, stimuli-responsive strategies and bionic modifications. It not only greatly improved delivery efficiency but also optimized the safety and bioavailability of MLT in vivo. This section reviews the different delivery strategies of MLT to improve its antitumor effect and biocompatibility (Fig. 4).

### Passive targeting

#### EPR effect

**Fig. 5:**
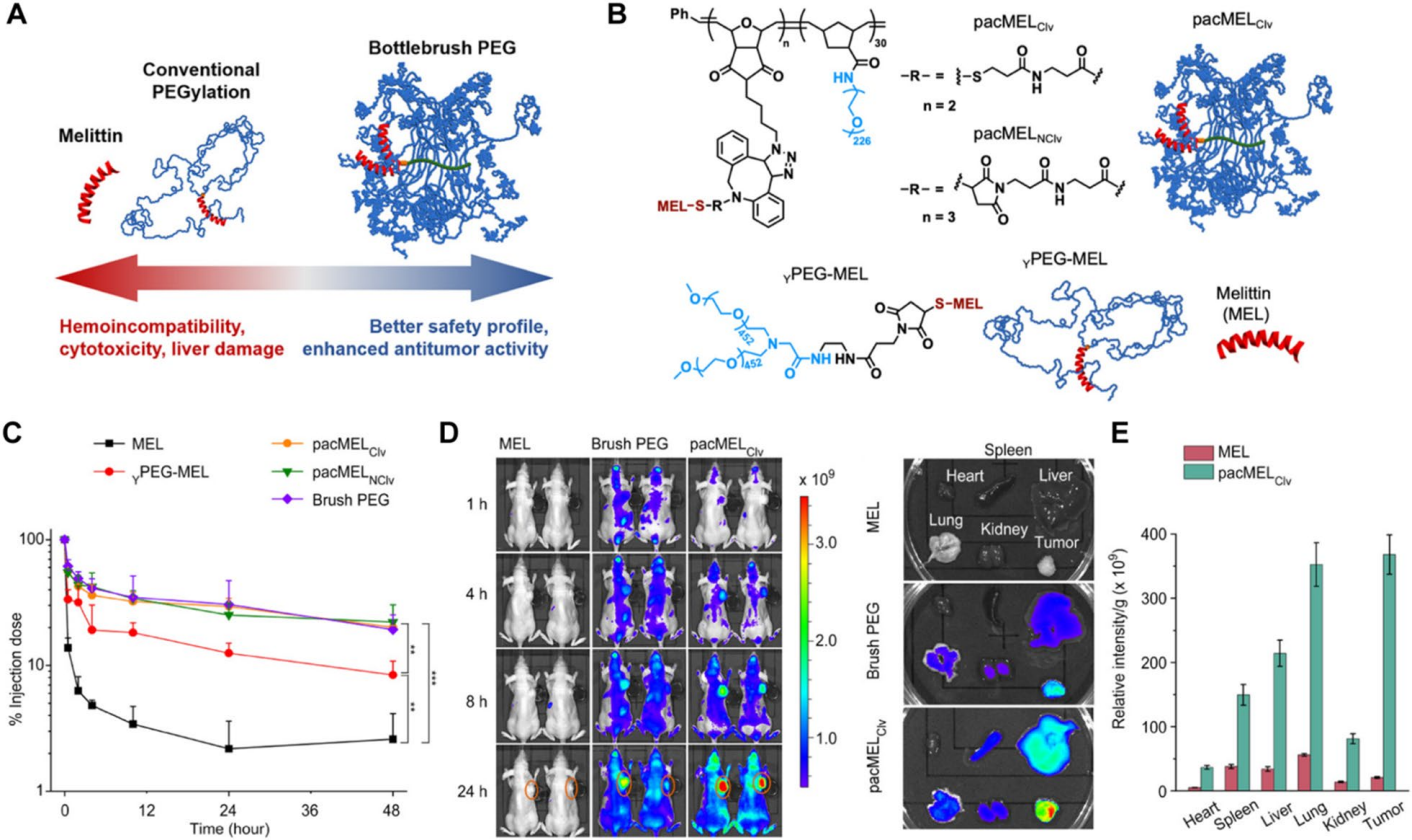
**A** Structural schematic diagram and biological properties of pacMEL_Clv_ and _Y_PEG-MEL. **B** Chemical structures and schematic illustrations of PEGylated MEL. **C** Plasma pharmacokinetics of MLT-containing samples and free bottlebrush polymer in C57BL/6 mice. **D** Near-IR imaging of BALB/c mice bearing NCI-H358 xenografts 24 h after i.v. injection with Cy5.5-labeled free MLT, pacMEL_Clv_, and bottlebrush polymer (Tumors are highlighted with orange circles). Ex vivo imaging of tumors and other major organs. **E** Biodistribution profile determined from image analysis. Reproduced with permission from [68]. Copyright 2021, American Chemical Society

Due to poor lymphatic drainage and the unique intraorgan pressures of tumors, nanoscale carriers can preferentially extravasate into the tumor site through leaky vessels [91]. As a main mechanism for passive tumor targeting, the enhanced permeability and retention (EPR) effect is a molecular weight-dependent phenomenon due to an increase in vascular permeability [92]. Various biomimetic NPs have been extensively designed as drug delivery systems that can be used to direct drug encapsulation or drug conjugation. These biomimetic NPs provide good biocompatibility to prevent them from being cleared from the body via the reticuloendothelial system (RES) [93]. In addition, owing to the leaky tumor vasculature and damaged lymphatic drainage, antitumor agents can also be more selectively accumulated at tumor sites via the EPR effect in vivo [94–96]. Xu et al. designed a library containing 82 self-assembled nanoparticles (SNPs) based on β-cyclodextrin polymers and adamantane derivatives and screened eight different types of SNPs with different charges and hydrophobic properties to suppress the toxicity of MLT to normal cells [97]. Compared with small-sized spherical particles, nonspherical particles constructed with minimal curvatures and high aspect ratios exhibit tumbling and rolling dynamics under flow in blood circulation to evade phagocytosis [98]. Therefore, they can marginate toward vessel endothelial walls in circulation and infiltrate into tumor tissues through fenestrated vasculatures [99]. Moreover, the increase in the curvature of membranes favors the insertion efficiency and functionality of MLT for the same copolymer [100]. However, they are not suitable for systemic administration because the surface load of MLT cannot completely shield hemolysis. To address this deficiency, some researchers have constructed nanosized lipodisks with flat circular phospholipid bilayers to achieve codelivery of paclitaxel and MLT, which were functionalized with glycopeptide ^9^G-A7R. MLT was fully protected from proteolysis, and hemolysis was effectively reduced. Finally, it effectively enhanced the antiglioma effect and significantly prolonged the survival time of glioma-bearing mice [101]. In addition, the bottlebrush polymer can provide extraordinary steric shielding to the embedded MLT through the high-density arrangement of the polyethylene glycol (PEG) side chains, allowing the conjugate to reduce nonspecific interactions with various proteins and cells in circulation. Due to the enhanced passive targeting of tumor xenografts via the EPR effect, it not only significantly prolonged the blood circulation times but also exhibited a more favorable biodistribution profile. Thus, the novel form of PEGylated MLT exerted significant tumor-suppressive activity without hemolytic activity or liver damage [68] (Fig. 5).

#### Size-dependent targeting

**Fig. 6:**
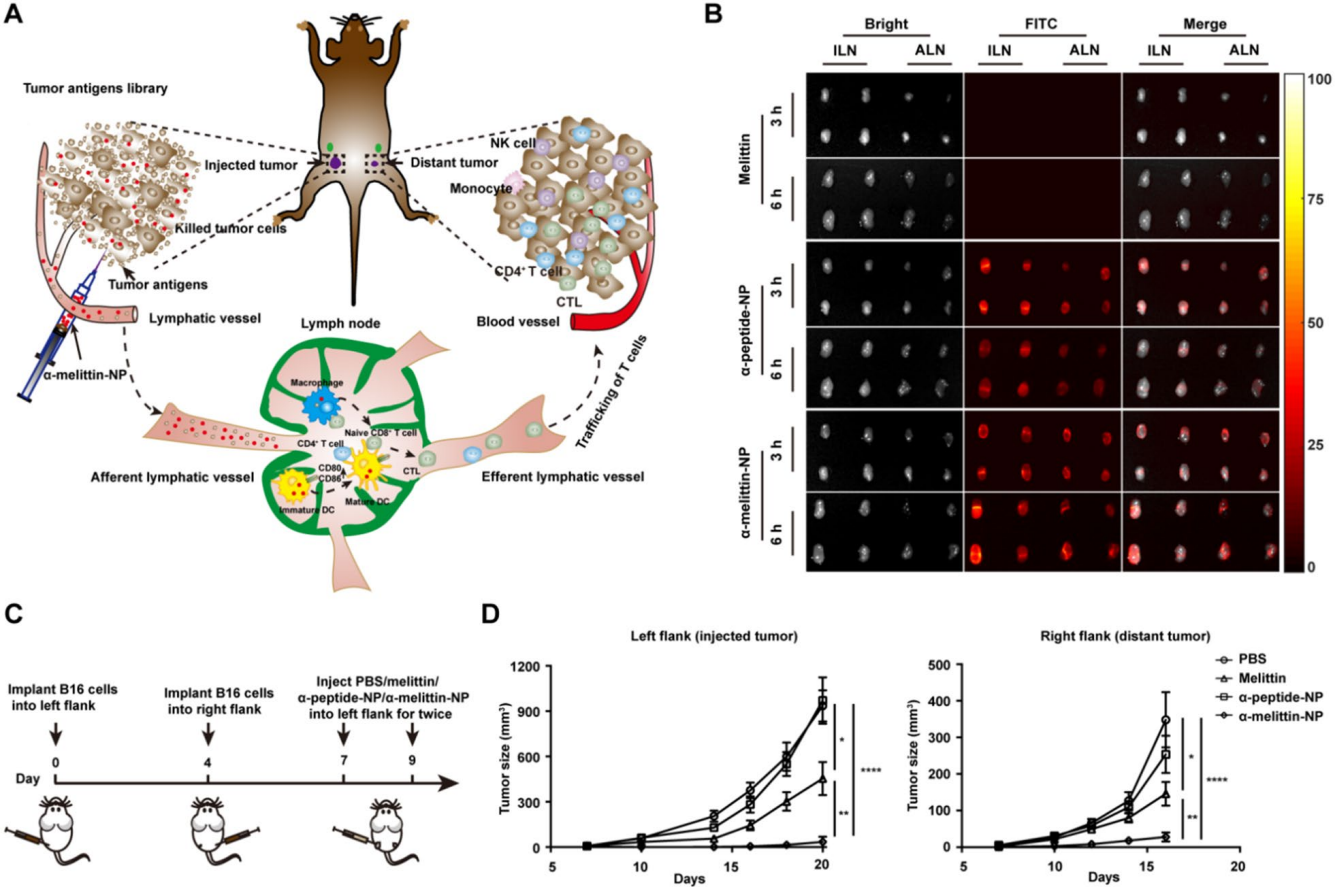
**A** Schematic description of the mechanism of the in situ vaccine effect induced by α-melittin-NPs.** B** Fluorescence images of excised LNs from C57BL/6 mice subcutaneously injected with 20 nmol FITC-melittin, FITC-α-peptide-NPs, and FITC-α-melittin-NPs (quantification was based on the FITC content).** C** Scheme of the bilateral flank tumor model established with B16F10 cells and the treatment scheme of different drugs.** D** Tumor growth of the injected tumor and distant tumor**.** Reproduced with permission from [57]

Size is one of the important physicochemical properties of nanocarriers and significantly affects blood circulation and biological distribution. Nanocarriers with diameters less than 6 nm are easily cleared by the liver. In contrast, small drugs less than 100 nm can freely pass through the vascular wall of normal and tumor tissues, thus lacking system selectivity and causing poor selectivity and toxic side effects. Diameters greater than 200 nm are captured and cleared by the liver prior to entering systemic circulation [101]. NP sizes ranging from 100 to 200 nm preferentially leak into tumor tissues through the permeable tumor vasculature, and then they might be retained in the tumor due to reduced lymphatic drainage [93, 102]. LNs are important secondary lymphoid organs that are strategically distributed throughout the body and form the body’s systemic immune surveillance for the immune response [103]. Meanwhile, lymph metastasis is a vital pathway of cancer cell dissemination, indicating that LNs are a potential target for cancer immunotherapy [55]. Nevertheless, current therapeutics for lymph metastasis are largely limited by the weak targeting and penetration capacity of drugs within metastatic LNs [104]. It has been found that the transport of drugs from subcutaneous tissue to LNs highly depends on particle size. After interstitial administration, small molecules or moderately sized macromolecules (< 16–20 kDa or 10 nm) are absorbed primarily via the blood capillaries. Due to reduced diffusion and convection through the interstitium, particles > 100 nm in size are also poorly transported into LNs, whereas macromolecules (20–30 kDa or 10–100 nm) mainly enter lymphatic vessels [105]. Therefore, NPs with small sizes, especially when the diameter is less than 30 nm, might offer a new avenue for targeting and even treating LN metastasis [106]. Previously, we loaded MLT onto a high-density lipoprotein-mimicking peptide-phospholipid scaffold to form an MLT-lipid NP with a particle size of approximately 20 nm. α-MLT tightly interacted with phospholipids to deeply and efficiently bury the cationic amino acids of MLT, thus remarkably reducing the hemolytic activity [107]. Consequently, α-melittin-NPs, as an optimal LN-targeted nanovaccine, could efficiently drain into lymphatic capillaries and LNs to activate APCs in LNs. Subsequently, the systemic humoral and cellular immune responses elicited by α-melittin-NPs resulted in the elimination of primary tumors and distant tumors in a bilateral flank B16F10 tumor model [57] (Fig. 6).

#### Endocytosis

Endocytosis is a complex but essential process whereby cell surface substances from the extracellular environment are packaged, sorted and internalized into cells, such as proteins, lipids and fluid. It has also been regarded as a critical cellular transport mechanism for the internalization of different NPs into cancer cells [108]. Several possible routes were found to participate in the uptake of the exogeneous NPs, including caveolin-mediated endocytosis and clathrin-caveolin-independent endocytosis [109]. Recently, Daniluk et al. reported that MLT in complex with graphene could be taken up by cells via caveolin-dependent endocytosis to induce oxidative stress inside MDA-MD-231 cells [110]. When oxidative stress persists or exceeds a certain level, it causes oxidative damage to DNA and lipids, thereby potentially initiating cell death by apoptosis and inhibiting malignant progression. Therefore, passive targeting is also expected to be achieved by the endocytic process of the NPs.

### Active targeting

#### Fusion peptide

Fusion peptides are one of the more commonly used derivation methods to achieve and optimize tumor-specific targeting ability by conjugating targeting ligands with MLT. Previously, Shin et al. engineered gelonin-MLT fusion proteins through chemical conjugation and genetic recombination methods [111]. It could bind and be internalized by tumor cells by utilizing the pore structures generated by MLT, eventually leading to greater cytotoxic effects and significant tumoricidal effects. However, it should be noted that the universal activity of MLT could also provoke severe side effects after systemic administration. Therefore, it is still necessary to explore a new method or drug delivery system to offer safe and effective administration. One study reported that a fusion protein containing VEGF165 and MLT (VEGF165-MLT) can inhibit tumor growth because it selectively targets tumor cells that overexpress VEGFR-2 [112]. In addition, Sun et al. used the urokinase-type plasminogen activator (uPA) cleavage site as a peptide linker to conjugate disintegrin and MLT (disintegrin-linker-MLT, DLM) [113]. The fusion peptide DLM selectively targeted uPAR on tumor cell surfaces with high efficiency and accuracy. Subsequently, MLT and disintegrin domains were released when the DLM reached the target cell and were cleaved by uPA. Therefore, DLM exhibited strong cytotoxicity against uPAR-expressing A549 lung cancer cells while confining the hemolytic activity of MLT and increasing the safety of delivery.

**Fig. 7:**
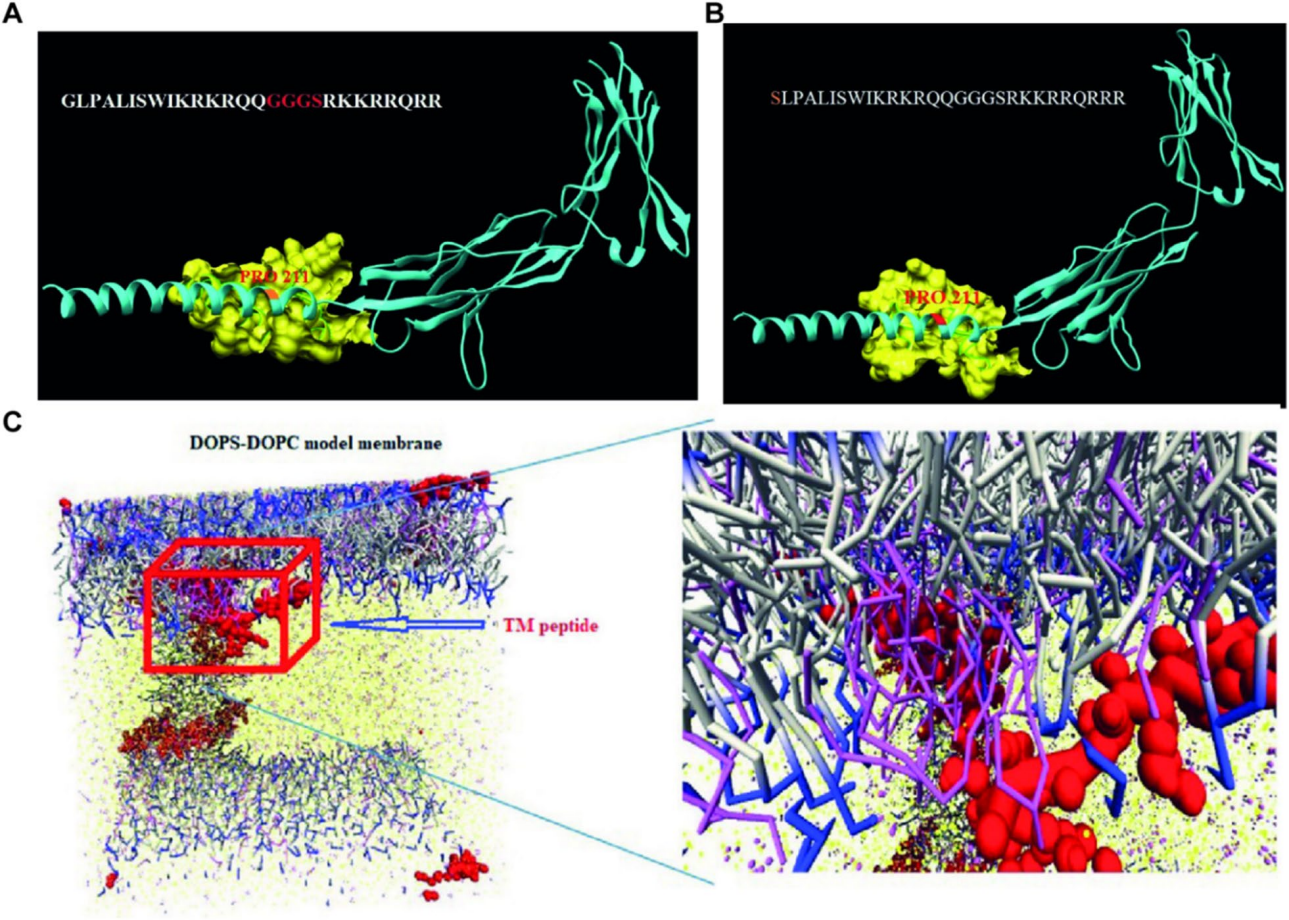
**A** The hybrid peptide indicated specific binding in a defined location (proline 211) by fusing MLT and TAT sequences by a GGGS linker. The blue, yellow and red colors are related to the CD147 protein, peptide and binding site (proline 211), respectively.** B** TM was as follows (SLPALISWIKRKRQQGGGSRKKRRQRRR), and the specific binding was confirmed by cluspro docking. The blue, yellow and red colors are related to the CD147 protein, TM peptide and binding site in proline 211, respectively.** C** Coarse-grained simulation of the TM peptide interaction with the DOPC/DOPS model membrane after 1500 ns (yellow = water, red = protein, blue, pink, light gray = lipid). Reproduced with permission from [118]. Copyright 2023, Elsevier

In addition, several studies have shown that MLT itself possesses the targeting ability to tumor cells and immune cells [114, 115]. Ciara et al. reported that MLT could induce potent and highly selective cell death in HER2-enriched and triple-negative breast cancer with negligible effects in normal cells by interfering with the phosphorylation of EGFR and HER2 receptors. Fusion engineering of an RGD motif further enhanced targeting of MLT to breast cancer cells and significantly increased the therapeutic window [115]. A previous study revealed that MLT only reduces M2-like TAMs in tumor tissue without affecting splenic macrophages. Therefore, MLT might be a promising therapeutic agent for targeting M2-like TAMs [116]. On this basis, Lee et al. designed the novel fusion peptide MEL-dKLA containing the pro-apoptotic peptide dKLA, which could specifically induce apoptosis of CD206+M2-like TAMs in the murine tumor stroma without affecting M1-like macrophages and other leukocytes [117]. It was proven that MEL-dKLA effectively inhibited tumor progression and angiogenesis within a noncytotoxic dose of MLT. Moreover, MLT blocked the MMP pathway and inhibited caspase activity by intervening in the interaction between CD147 and its ligand cyclophilin A (CypA). The latest research reported that the C-terminal portion of MLT was fused to a TAT cell-penetrating peptide by the GGGS linker to construct the TM peptide, which could be bound to CD147 with sufficient binding energy to block the cyclophilin A/CD147 interaction [118] (Fig. 7). Therefore, the TM fusion peptide is regarded as a therapeutic candidate to halt tumor progression and coronavirus disease 2019 (COVID-19) infection.

#### Aptamer–based conjugation

Aptamers are oligonucleotides that can effectively recognize and bind to a wide range of targets ranging from small molecules to whole cells without toxicity and immunogenicity. A previous study reported a targeted drug delivery system through the covalent conjugation of MLT to the AS1411 aptamer, which can selectively bind to the extracellular matrix protein nucleolin. It could be specifically internalized and delivered to several nucleolin-expressing tumor cells while reducing the hemolytic activity and cytotoxic effects of MLT on normal cells [119]. Furthermore, calcium carbonate NPs modified with mucin 1 (MUC1) dimer aptamers could also deliver epirubicin and MLT to cancer cells overexpressing MUC1 glycoproteins. The complexes exhibited strong synergistic cytotoxicity as well as a better effect on controlling tumor growth than monotherapy [120]. Our own unpublished experiments also showed that MLT could bind to the negatively charged aptamer of Lc09, which specifically targets K7M2 murine osteosarcoma cells. Electrostatic interactions contribute to reduced hemolytic activity, while MLT displayed a more pronounced antitumor effect due to the targeted killing of K7M2 cells.

#### Specific receptor–ligand binding

**Fig. 8:**
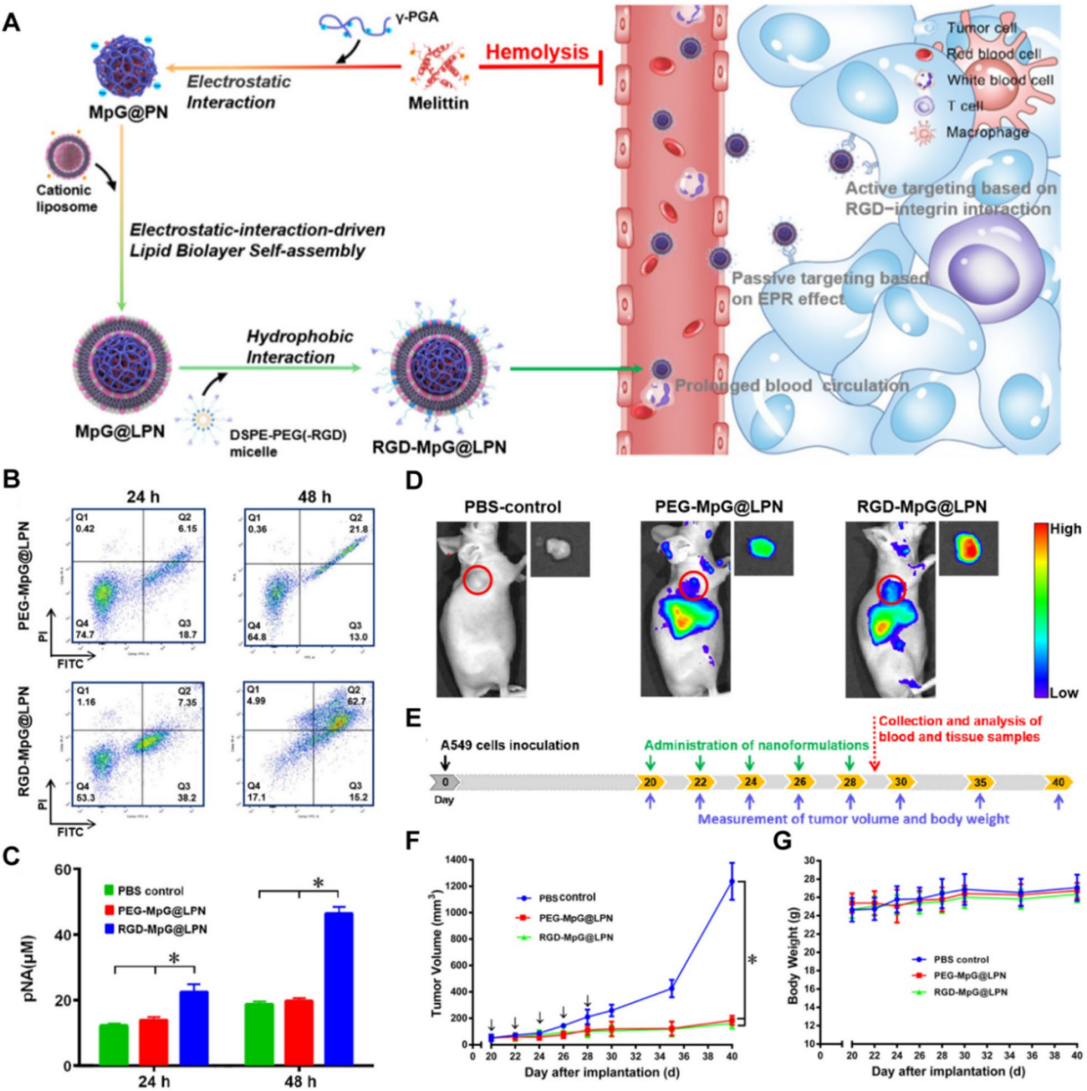
**A** Schematic illustration of the fabrication and tumor-targeted therapy of MpG@LPN.** B** Cell apoptosis/necrosis analysis of A549 cells treated with PEGMpG@LPN and RGD-MpG@LPN by Annexin V/PI-FACS.** C** Caspase-3 activities in A549 cells treated with PEG-MpG@LPN and RGD-MpG@LPN.** D** Images of tumor-bearing nude mice at 6 h after the administration of DiR-labeled PEG-MpG@LPN and RGD-MpG@LPN and ex vivo tumors at 24 h postinjection. **E** Flowchart of the antitumor experiment in vivo. **F** Tumor growth of A549 xenograft mice treated with PBS, PEG-MpG@LPN, and RGD-MpG@LPN. **G** Changes in the body weight of tumor-bearing mice in different groups. Reproduced with permission from [128]. Copyright 2021, American Chemical Society

Tumor cells over express various receptors on the cell membrane, providing an appealing therapeutic target. Different targeted molecules act as “missiles” to deliver drugs with antitumor activity and achieve selective targeting through the high affinity between ligands and receptors. For example, hyaluronic acid (HA) not only increases the accumulation of NPs in the CD44-expressed tumor region but also results in the release of therapeutic agents in the TME by hyaluronidase-mediated degradation of HA [121]. Therefore, the insertion of HA moieties as an effective targeting element within NP formulations is a promising strategy for tumor treatment [122, 123]. Jia et al. found that chlorin e6 (Ce6) could form a stable nanocomplex with MLT and effectively shield the hemolytic activity of MLT [124]. Meanwhile, HA was coated on the surface of the nanocomplex for tumor targeting and reduced the cytotoxicity of MLT toward normal cells. Then, Ce6 could generate oxidative ^1^O_2_ upon laser irradiation to enhance tumor penetration and improve anticancer efficiency. In addition, folic acid (FA) receptors are well known as tumor-associated proteins and are expressed to a great extent on numerous solid tumors, indicating that FA is a desirable choice for active targeting. Motiei et al. developed an innovative FA-decorated multilayer polyelectrolyte nanocarrier containing LTX-315, MLT and miR-34a to selectively inhibit triple-negative breast cancer [125]. It was synthesized via layer-by-layer self-assembly of polycationic and polyanionic chains to achieve specific codelivery of MLT with miR-34a and increased smart death induction of MDA-MB-231 cells by 54% after 48 h. Arginine-glycine-aspartic acid (RGD) is a peptide that specifically binds fibronectin and integrin αvβ3, which can potentiate tumor angiogenesis and metastasis [126]. To date, various RGD-based targeting approaches have been intensively investigated for precise imaging and tumor interventions [127]. Recently, researchers developed MLT-loaded lipid-coated polymeric nanoparticles (MpG@LPN) with a MEL/poly-γ-glutamic acid (MpG) NP inner core, a lipid membrane middle layer, and a hydrophilic PEG-RGD molecule for stealth and active targeting. MpG@LPN not only effectively prevented the leakage of MLT to eliminate subsequent hemolysis and nonspecific cytotoxicity but also remarkably inhibited tumor growth via the specific binding of the RGD peptide to integrin ανβ3 and selective apoptosis-inducing ability [128] (Fig. 8). Overall, receptor–ligand mediated targeting can promote maximum drug accumulation in tumor tissue to enhance the therapeutic value of MLT while decreasing the risks of adverse effects and toxicity to normal tissues.

### Stimulus-responsive targeting

#### Enzyme response

**Fig. 9:**
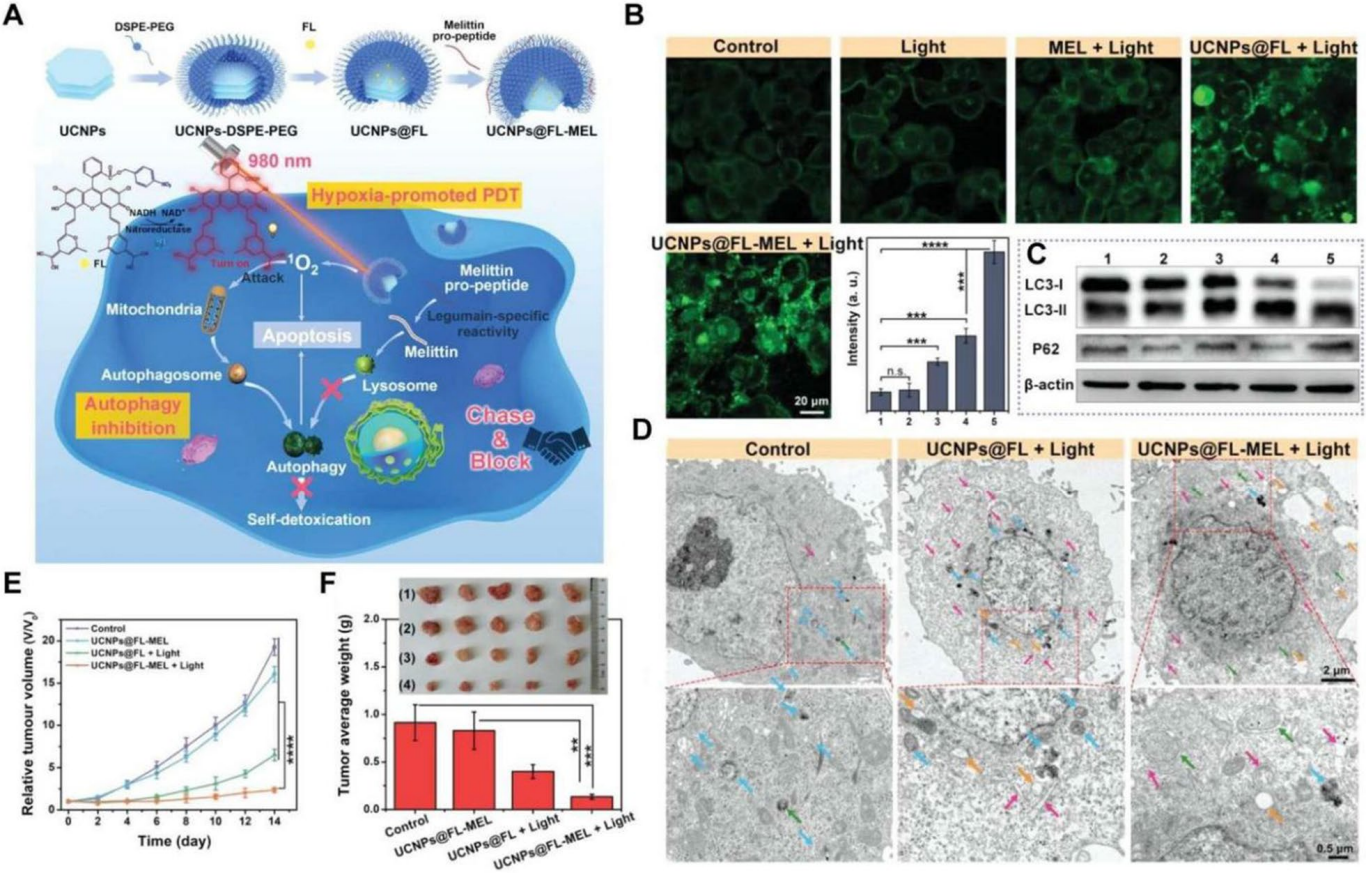
**A** Schematic illustration of the synthesis of UCNPs@FL-MEL and the mechanism for combination therapy of hypoxic tumors. **B** Confocal fluorescence images of HeLa cells stained with Ad-GFP-LC3B after various treatments and the statistics of the corresponding fluorescence intensity values.** C** Changes in key autophagy-related proteins, including P62, LC3-II and LC3 were investigated by Western blot in HeLa cells.** D** The cellular autophagy levels of HeLa cells after various treatments were observed by Bio-TEM images. The pink, green, blue and orange arrows represent the phagophore, autophagosome, autolysosome, and vacuoles, respectively. **E** Tumor volume changes in tumor-bearing Balb/c mice after different treatments. **F** Images of representative excised tumors from different groups after 14 days of treatment and the corresponding mean weight of the tumors. Reproduced with permission from [137]. Copyright 2023, Wiley

Most invasive diseases are characterized by elevated levels of some secreted or membrane-bound enzymes that possess outstanding capabilities in biorecognition and catalysis [129, 130]. Therefore, specific spatiotemporal control of drug release can be achieved using certain nanocarriers or delivery strategies capable of responding to disease-specific enzymes [131]. Legumain, a novel asparaginyl endopeptidase, can specifically recognize and cleave the COOH terminus of the substrate peptide (NH2-alanine–alanine–asparagine-COOH, AAN) [132]. Based on this, a tumor-activated size-enlargeable bioinspired lipoprotein of oxaliplatin (TA-OBL) was designed by integrating a legumain-sensitive MLT conjugate with drugs to enhance the intratumoral permeation and cancer cell accessibility of drugs. TA-OBL produced robust antitumor immune responses in vivo and its combination with immune checkpoint blockades demonstrated notable therapeutic benefits with delayed tumor growth and an extended survival rate [45]. There are also reports that have indicated that MLT can cross the intestinal epithelial barriers lining the gastrointestinal (GI) tract to improve oral drug bioavailability, suggesting its potential as an absorption enhancer without cytotoxicity [133, 134]. In addition, Wang et al. developed a legumain-activable MLT-decorated polymeric nanovehicle (LPN) to enhance the oral delivery efficiency of sorafenib for gastric cancer chemotherapy [135]. In this study, LPN was effectively absorbed across the GI barriers into the circulation system due to the enhancement effects of MLT on preferential accumulation and deep penetration at tumor sites, resulting in notable inhibition of tumor growth through LPN-mediated combinational photothermal-chemotherapy. However, it is difficult to completely control the release of drugs with high efficiency based on the enzyme response alone [136]. Therefore, the combination of enzyme response with on-demand therapeutic delivery would effectively improve utilization efficiency. The latest study reported a synergistic therapeutic system based on hypoxic-sensitive photosensitizers and MLT, which possessed dual activation of PDT by near infrared (NIR) and the TME. Concretely speaking, owing to overexpressed nitroreductase in the hypoxic tumor region, UCNPs@FL-MEL cleverly transformed the "Achilles heel" of tumor hypoxia into an activating factor of the hypoxia-sensitive organic photosensitive molecule. Simultaneously, MLT was hydrolyzed specifically by legumain in tumor cells and inhibited the formation of autophagic lysosomes [137] (Fig. 9). In addition, Zhang et al. developed a genetically engineered vesicular antibody-melittin (VAM) drug delivery nanoplatform that could target glypican 3-positive hepatocellular carcinoma for multimodal synergetic cancer therapy. Remarkably, VAM exhibited significant antitumor effects when combined with chemotherapy or SDT without the generation of severe side effects through intravenous administration [138]. The bioactive nanoMelittin could be released specifically in an MMP14-responsive manner at tumor sites and then form pores in membranes and disturb phospholipid bilayers of tumor cell membranes. Different antitumor agents could also be loaded on this smart nanoplatform, especially sonodynamic therapy agents, to improve the immunomodulatory effect of nanoMelittin and trigger a robust antitumor immune response.

#### Redox sensitivity

**Fig. 10:**
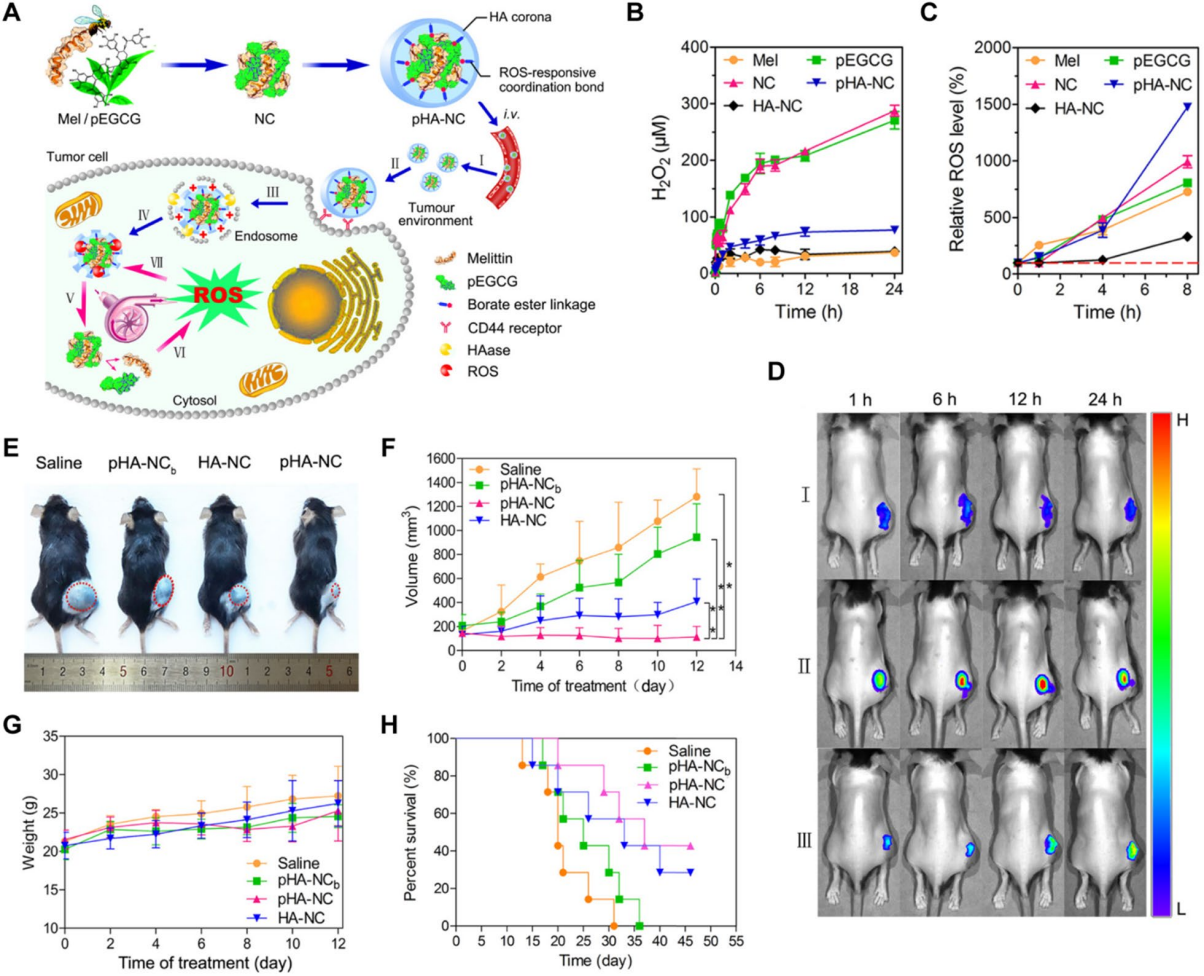
**A** Schematic illustration of a multipronged nanocomplex (pHA-NC) preparation and the mechanism for ROS self-sufficient oxidation therapy of cancers.** B** H_2_O_2_ production from different formulations in pH 7.4 PBS solution.** C** Relative ROS levels in B16F10 cells incubated with different formulations and times were measured by flow cytometry after DCFH-DA staining.** D** Fluorescence imaging of ROS generation in differently treated B16F10 tumor-bearing mice at 1, 6, 12 and 24 h, which were intratumorally injected as an indicator of ROS with DCFHDA (2.5 mg/kg) before intravenous injection of saline (I), pHA-NC (II) and HA-NC (III) at a dose of 2.5 mg/kg MLT. **E** Representative images of B16F10 xenograft tumors in mice after different treatments on day 12. **F** Tumor growth curves of mice after different treatments (n = 5). **G** Body weight changes in B16F10 tumor-bearing mice during treatments (n = 5). **H** Animal survival rates after different treatments. Reproduced with permission from [144]. Copyright 2018, American Chemical Society

Abnormal cancer metabolism and the inflammatory microenvironment result in substantially greater differences in redox homeostasis between solid tumors and healthy tissues, such as elevated levels of ROS and an abnormal disulfide redox state [139]. Accordingly, a redox-responsive NP technology is emerging as a triggering mechanism for drug activation. Cheng et al. developed a poly[(2-(pyridin-2-yldisulfanyl) ethyl acrylate)-co-[poly(ethylene glycol)]] polymer derivative that could form a complex with MLT through electrostatic forces [140]. It entered cancer cells through lactobionic acid-mediated endocytosis. Subsequently, the high redox potential of the intracellular environment could break the disulfide bonds and trigger the release of MLT intracellularly, ensuring that MLT would not be released prematurely before reaching the targeted tumor sites to prevent the occurrence of hemolysis. Compared to normal tissues, tumors have a profuse metabolism and a great oxidative state, which increases the levels of mitochondrial ROS generation [141]. Meanwhile, the ROS levels in tumor cells and in normal cells differ by hundreds or even thousands of times. Therefore, ROS-activable NPs based on a series of ROS-responsive cleavable chemical structures, such as diselenide and thioether bonds, have gradually become an important therapeutic strategy [142, 143]. Qiao et al. reported that the complexation of MLT and poly-epigallocatechin gallate (pEGCG) can be covered with phenylboronic acid-derivatized HA (pHA) via the ROS-responsive boronate ester coordination bond to produce the ROS cascade-amplifying nanodevice pHA-NC. Upon delivery to the tumor region by the EPR effect and undergoing receptor-mediated endocytosis into cancer cells, the inner cores of pHA-NC would be exposed once the pHA corona was degraded by hyaluronidase. Meanwhile, the elevated ROS level in the tumor cytoplasm disrupted the boronate ester bond to trigger the release of drugs. Finally, both pEGCG and MLT, as natural pro-oxidants, synergistically amplified oxidative stress and prolonged ROS retention in cancer cells, thereby significantly inhibiting tumor progression with minimal side effects [144] (Fig. 10).

#### pH sensitivity

**Fig. 11:**
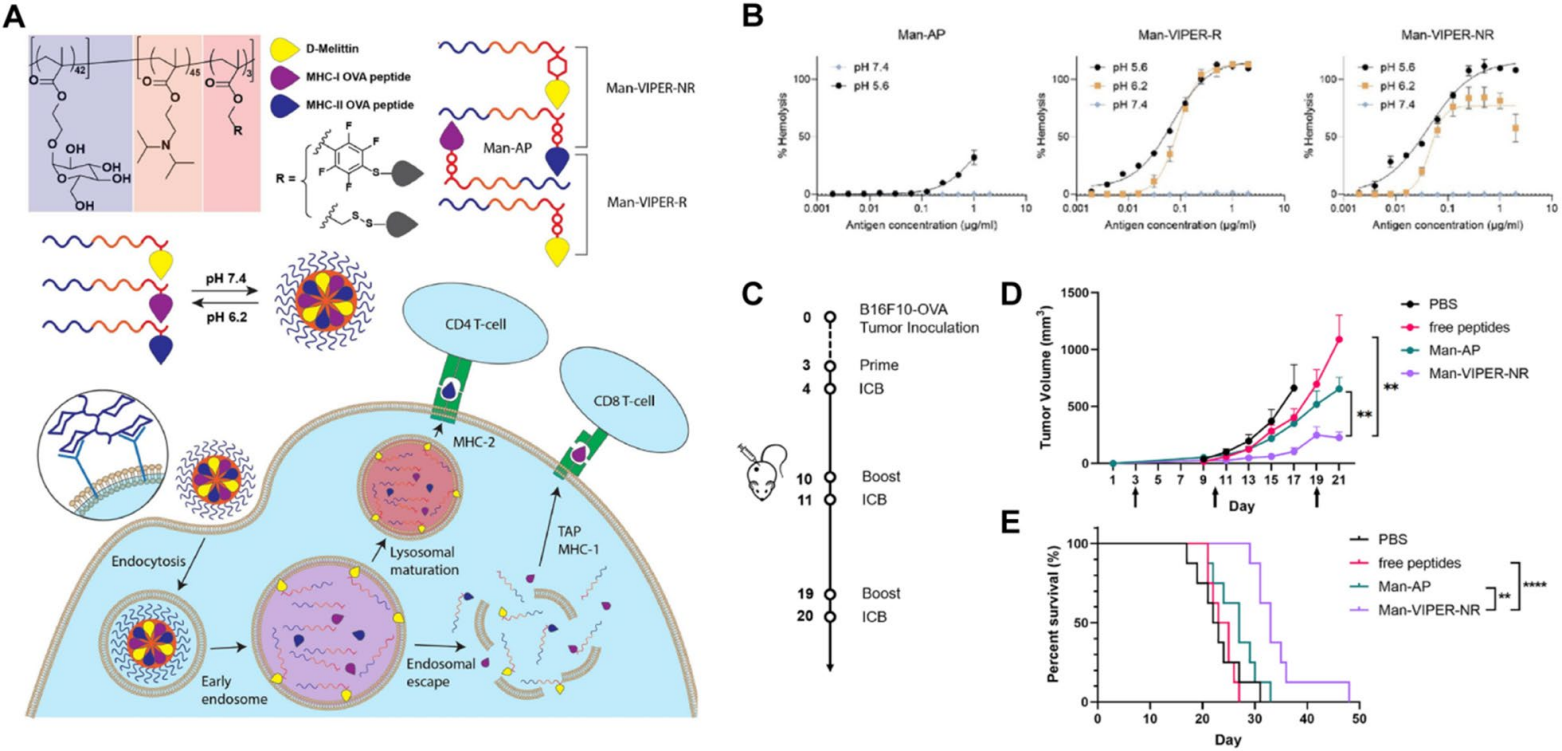
**A** Schematic illustration of the preparation of Man-VIPER-R and Man-VIPER-NR delivery systems, as well as their function in vivo. The formulations of self-assembled micelles are preferentially endocytosed via the mannosylated hydrophilic segment anddisassembled upon endosomal maturation.Then, it results in endosomal disruption and cross-presentation of MHC-I epitopes or lysosomal maturation and presentation of MHC-II epitopes to their own respective T-cell subsets.** B** Hemolytic activity of different micelles against human RBCs at pH 5.6, pH 6.2 and pH 7.4.** C** Scheme of the B16F10-OVA tumor model and treatment scheme of different drugs. Mice were vaccinated on days 3, 10, and 19 and treated with ICB on days 4, 11, and 20.** D** Tumor growth curve of the tumor-bearing mice from the different treatment groups (n = 8). **E** Kaplan‒Meier survival curve from the different treatment groups. Reproduced with permission from [146]. Copyright 2023, Elsevier

The hypoxic environment in solid tumors promotes glycolysis and produces a slightly acidic microenvironment versus normal tissue. Therefore, NPs with delicate surface characteristics could remain inactivated in blood circulation but respond to pH changes in the TME, thus rapidly converting to an activated state to achieve specific tumor therapy. In a previous study, 2,3-dimethylmaleic anhydride (DMMA) was proposed to modify and convert the original positive charge of the active amino groups in MLT to develop an ultra pH-sensitive MLT-DMMA for targeted antitumor therapy. It exhibited sufficient safety in the blood circulation, but once it arrived in tumor tissue, the pH labile imide bond would be cleaved under slightly acidic tumoral conditions to exert timely antitumor activity in the tumor lesion [145]. In addition, Lv S et al. constructed a novel virus-inspired polymer for endosomal release (VIPER) platform by conjugating D-melittin to an ultra pH-sensitive polymer, which shields MLT at physiological pH and rapidly unsheaths MLT at endosomal pH [42]. To further surmount the systemic and cellular barriers of the VIPER platform, their lab focused on developing a powerful peptide cancer vaccine platform that exhibited the desired pH-responsive property for LN-targeted antigen delivery. In this study, the mannosylated VIPER formulation could induce efficient endosomal release and MHC-I antigen presentation upon endocytosis and acidification. Ultimately, this platform significantly inhibited tumor growth and prolonged survival in an aggressive melanoma model by inducing potent T-cell responses against specific tumor antigens [146] (Fig. 11).

#### Temperature sensitivity

Due to their excellent spatiotemporal controllability, temperature-sensitive NPs for targeted drug delivery have received much attention in cancer treatment [147]. On the one hand, drug release shows drastic changes in response to external changes in temperature. On the other hand, mild hyperthermia has been shown to increase the perfusion, cellular permeability, and sensitivity of tumor cells to chemotherapy or radiotherapy. In a recent study, Koide et al. described a novel approach that employed a temperature-responsive polymer NP based on poly(N-isopropylacrylamide) (pNIPAm) for the selective delivery of MLT [148]. It had a higher affinity for loaded MLT at physiological temperatures but lost its affinity and rapidly released MLT at tumor sites upon cooling below its lower critical solution temperature (LCST). The cooling-induced release of MLT from pNIPAm-based copolymer nanoparticle 6 (Peg-NP6) effectively inhibited the growth of tumors while minimizing peripheral damage to cells in other organs. This research was the first example of site-specific release of a macromolecular drug by local cooling for cancer therapy, opening a new field of temperature-responsive polymers for in vivo biomedical applications.

## Conclusion and prospects

Although MLT has the potential to overcome tumor heterogeneity and resistance to chemotherapy, the use of free MLT is limited due to adverse side effects. Recent years have witnessed tremendous progress in the antitumor mechanisms and targeted delivery strategies of MLT, thus providing a possibility for clinical application. At the same time, further exploration of the antitumor molecular mechanisms of MLT will contribute to providing clinical insights and a theoretical foundation for optimizing targeted synergistic therapy.

MLT was originally separated and purified from honeybee (*Apis mellifera*) venom, which contains a variety of active substances, including peptide enzymes, biologically active amines and some nonpeptide components. Interestingly, differences between different honey bee species affect the compositions and contents. Recently, Maitip et al. suggested that different honey bee species have different MLT amino acid compositions [149]. There was, of course, a disparity in activity between the crude MLT from venom and synthetic MLT. In addition, one of the bottlenecks when studying toxins from small or rare venomous species is the difficulty of obtaining large amounts of venom and purified toxins. How to optimize the reasonable extraction or synthesis process of MLT to realize large-scale mass production is urgently needed but still very challenging. As the third wave for peptide synthesis, liquid-phase peptide synthesis (LPPS) allows the large-scale production of peptides and reduces the energy and material consumption costs. Therefore, it is possible to obtain MLT in kilograms or tons through LPPS. Additionally, susceptibility and immunogenicity, which are directly linked to biopharmaceutical degradation in clinical applications, are also important factors to be considered. Venom immunotherapy is regarded as the only treatment approach to prevent anaphylactic reactions. Interestingly, in a recent study, low-dose α-melittin-NPs suppressed the DC activation caused by allergens and the proliferation and activation of allergen-specific T cells, leading to efficient immunosuppression against T cell-mediated immune responses in allergic contact dermatitis [150]. In addition, it was shown that utilizing D-melittin instead of L-melittin diminishes antibody generation and immunogenicity [151]. These results may provide new and promising strategies to avoid the allergic risk of MLT. Notably, repeated administration of NP carriers conjugated with biologically active drugs is likely to trigger a host immune response and elicit antibodies against the carrier itself. In addition, PEG-related immunological issues have received considerable attention. The presence of anti-PEG antibodies can result in rapid blood clearance of PEGylated drugs, even leading to life-threatening hypersensitivity reactions. Reducing the immunogenicity of biologics contributes to the production of anti-carrier antibodies. Therefore, some biomimetic nanomedicines and carrier-free systems prepared with pure cargos without involving any organic or inorganic carriers have the potential to avoid immunogenicity and potential toxicity.

Although various MLT-based antitumor strategies have shown great potential in eliciting robust antitumor effects, almost all of them are still in their infancy. To exert the biological properties of MLT more effectively and safely, it is critical to design and develop tunable and predictable delivery strategies as well as choose appropriate antitumor combination strategies to realize its full potential in the field of tumor therapy. Currently, gene therapy based on nanotechnology holds great promise for treating diseases ranging from inherited disorders to tumors. Targeted delivery of plasmid DNA encoding MLT to tumor cells might achieve better therapeutic potential. More importantly, there is no need to consider the issues of MLT sources through genetic modification.

## Data Availability

The data that support the findings of this study are available from the authors upon reasonable request. The data shown in this article can be obtained by request.

## Abbreviations

MLT: Melittin
ΔΨm: Mitochondrial membrane potential
ERK: Extracellular Signal-Regulated Kinase
FAK: Focal adhesion kinase
SDF-1α: Stromal cell-derived factor 1α
CXCR4: Monoclonal antibody to Chemokine C-X-C-Motif receptor 4
JAK: Janus kinase
STAT: Signal transducer and activator of transcription
PI3K: Phosphoinositide 3 kinase Akt Protein Kinase
NF-κB: Nuclear factor kappa-B
MAPK14: Mitogen activated protein kinase 14
c-MYC: Cancer-MYC
NOD: Nucleotide-binding oligomerization domain
FITC: Fluorescein Isothiocyanate
CD44: Cluster determinant 44
ICB: Immune checkpoint blockade
MHC-I: Major histocompatibility complex-I
Dvl: Dishevelled
GSK3β: Glycogen synthase kinase 3 beta
mTOR: Mammalian target of rapamycin
RBC: Red blood cell
AP-1: Activating protein-1
tcf-1: Transcription factor T cell factor 1
LDH: Lactate dehydrogenase
CTL: Cytotoxic T lymphocyte
P2RX7: Recombinant purinergic receptor P2X, ligand gated ion channel 7
F IX: Coagulation factor IX
F IIa: Coagulation factor IIa
F X: Coagulation factor X
F XI: Coagulation factor XI
t-PA: Tissue-type plasminogenactivator
B2R: Bradykinin B2 receptor
PLT: Platelet
PAF: Platelet activating factor
PAR: Protease-activated receptor
IgE: Immunoglobulin E

## References

[1] Kwon NY, Sung Sung H, Ferlay J, Siegel RL (2021). Global cancer statistics 2020: GLOBOCAN estimates of incidence and mortality worldwide for 36 cancers in 185 countries. CA Cancer J Clin.

[2] Zugazagoitia J, Guedes C, Ponce S, Ferrer I, Molina-Pinelo S, Paz-Ares L (2016). Current challenges in cancer treatment. Clin Ther.

[3] Avci FG, Akbulut BS, Ozkirimli E (2018). Membrane active peptides and their biophysical characterization. Biomolecules.

[4] Zhou J, Wan C, Cheng J (2021). Delivery strategies for melittin-based cancer therapy. ACS Appl Mater Interfaces.

[5] Kwon NY, Sung SH, Sung HK, Park JK (2022). Anticancer activity of bee venom components against breast cancer. Toxins.

[6] Zhou Y, Zhang S, Chen Z (2020). Targeted delivery of secretory promelittin via novel Poly(lactone-co-β-amino ester) nanoparticles for treatment of breast cancer brain metastases. Adv Sci.

[7] Zhou Y, Ye T, Ye C (2021). Secretions from hypochlorous acid-treated tumor cells delivered in a melittin hydrogel potentiate cancer immunotherapy. Bioact Mater.

[8] Han E, Kim D, Cho Y, Lee S, Kim J, Kim H (2023). Development of polymersomes co-delivering doxorubicin and melittin to overcome multidrug resistance. Molecules.

[9] Guo Y, Zhang X, Wang SZ, Feng HH, Wu SY, Wu FG (2023). Metal-phenolic network-facilitated “foe-to-friend” conversion of melittin for cancer immunotherapy with boosted abscopal effect. Research.

[10] Mohammed ER, Elmasry GF (2022). Development of newly synthesised quinazolinone-based CDK2 inhibitors with potent efficacy against melanoma. J Enzyme Inhib Med Chem.

[11] Jirawatnotai S, Hu Y, Michowski W (2011). A function for cyclin D1 in DNA repair uncovered by protein interactome analyses in human cancers. Nature.

[12] Mansour GH, El-Magd MA, Mahfouz DH (2021). Bee venom and its active component Melittin synergistically potentiate the anticancer effect of sorafenib against HepG2 cells. Bioorg Chem.

[13] Tipgomut C, Wongprommoon A, Takeo E, Ittiudomrak T, Puthong S, Chanchao C (2018). Melittin induced G1 cell cycle arrest and apoptosis in chago-K1 human bronchogenic carcinoma cells and inhibited the differentiation of THP-1 cells into tumour- associated macrophages. Asian Pac J Cancer Prev.

[14] Sun M, Wu Y, Zhou Z, Liu S, Mao S, Li G (2023). Co-delivery of EGCG and melittin with self-assembled fluoro-nanoparticles for enhanced cancer therapy. Aging.

[15] Fahmy UA, Badr-Eldin SM, Aldawsari HM (2022). Potentiality of raloxifene loaded melittin functionalized lipidic nanovesicles against pancreatic cancer cells. Drug Deliv.

[16] Shi P, Xie S, Yang J (2022). Pharmacological effects and mechanisms of bee venom and its main components: recent progress and perspective. Front Pharmacol.

[17] Obeidat M, Al-Khraisat IF, Jaradat DMM (2023). Mellitin peptide quantification in seasonally collected crude bee venom and its anticancer effects on myelogenous K562 human leukaemia cell line. BMC Complement Med Ther.

[18] Rady I, Siddiqui IA, Rady M, Mukhtar H (2017). Melittin, a major peptide component of bee venom, and its conjugates in cancer therapy. Cancer Lett.

[19] Moghaddam FD, Mortazavi P, Hamedi S, Nabiuni M, Roodbari NH (2020). Apoptotic effects of melittin on 4T1 breast cancer cell line is associated with Up regulation of Mfn1 and Drp1 mRNA expression. Anticancer Agents Med Chem.

[20] Yu CL, Lee HL, Yang SF (2022). Protodioscin induces mitochondrial apoptosis of human hepatocellular carcinoma cells through eliciting ER stress-mediated IP3R targeting Mfn1/Bak expression. J Hepatocell Carcinoma.

[21] Peña-Blanco A, García-Sáez AJ (2018). Bax, Bak and beyond—mitochondrial performance in apoptosis. FEBS J.

[22] Ceremuga M, Stela M, Janik E (2020). Melittin-A natural peptide from bee venom which induces apoptosis in human leukaemia cells. Biomolecules.

[23] Yan R, Dai W, Wu R, Huang H, Shu M (2022). Therapeutic targeting m6A-guided miR-146a-5p signaling contributes to the melittin-induced selective suppression of bladder cancer. Cancer Lett.

[24] Soliman C, Eastwood S, Truong VK, Ramsland PA, Elbourne A (2019). The membrane effects of melittin on gastric and colorectal cancer. PLoS ONE.

[25] Moreno M, Giralt E (2015). Three valuable peptides from bee and wasp venoms for therapeutic and biotechnological use: melittin, apamin and mastoparan. Toxins.

[26] Liu M, Wang H, Liu L, Wang B, Sun G (2016). Melittin-MIL-2 fusion protein as a candidate for cancer immunotherapy. J Transl Med.

[27] Xu T, Cui T, Peng L, Kong S, Zou J, Tian X (2017). The anti-hepatocellular carcinoma activity of Mel-P15 is mediated by natural killer cells. Oncol Lett.

[28] Rocha MM, Dariva I, Zornoff GC (2022). A new therapeutic approach for bone metastasis in colorectal cancer: intratumoral melittin. J Venom Anim Toxins Incl Trop Dis.

[29] Fomitcheva-Khartchenko A, Kashyap A, Geiger T, Kaigala GV (2022). Space in cancer biology: its role and implications. Trends Cancer.

[30] Huh JE, Kang JW, Nam D (2012). Melittin suppresses VEGF-A-induced tumor growth by blocking VEGFR-2 and the COX-2-mediated MAPK signaling pathway. J Nat Prod.

[31] Shin JM, Jeong YJ, Cho HJ (2013). Melittin suppresses HIF-1α/VEGF expression through inhibition of ERK and mTOR/p70S6K pathway in human cervical carcinoma cells. PLoS ONE.

[32] El Bakary NM, Alsharkawy AZ, Shouaib ZA, Barakat EMS (2020). Role of bee venom and melittin on restraining angiogenesis and metastasis in γ-irradiated solid ehrlich carcinoma-bearing mice. Integr Cancer Ther.

[33] Aufschnaiter A, Kohler V, Khalifa S (2020). Apitoxin and its components against cancer, neurodegeneration and rheumatoid arthritis: limitations and possibilities. Toxins.

[34] Liu S, Yu M, He Y (2008). Melittin prevents liver cancer cell metastasis through inhibition of the Rac1-dependent pathway. Hepatology.

[35] Salimian F, Nabiuni M, Salehghamari E (2022). Melittin prevents metastasis of epidermal growth factor-induced MDA-MB-231 cells through the inhibition of the SDF-1α/CXCR4 signaling pathway. Cell J.

[36] Chen M, Rong R, Xia X (2022). Spotlight on pyroptosis: role in pathogenesis and therapeutic potential of ocular diseases. J Neuroinflamm.

[37] Zhang Z, Zhang Y, Xia S (2020). Gasdermin E suppresses tumour growth by activating anti-tumour immunity. Nature.

[38] Wang Q, Wang Y, Ding J (2020). A bioorthogonal system reveals antitumour immune function of pyroptosis. Nature.

[39] Vandebriel RJ, David CAW, Vermeulen JP, Liptrott NJ (2022). An inter-laboratory comparison of an NLRP3 inflammasome activation assay and dendritic cell maturation assay using a nanostructured lipid carrier and a polymeric nanomedicine, as exemplars. Drug Deliv Transl Res.

[40] Martín-Sánchez F, Martínez-García JJ, Muñoz-García M (2017). Lytic cell death induced by melittin bypasses pyroptosis but induces NLRP3 inflammasome activation and IL-1β release. Cell Death Dis.

[41] Zhao Q, Feng H, Yang Z (2022). The central role of a two-way positive feedback pathway in molecular targeted therapies-mediated pyroptosis in anaplastic thyroid cancer. Clin Transl Med.

[42] Lv S, Sylvestre M, Song K, Pun SH (2021). Development of D-melittin polymeric nanoparticles for anti-cancer treatment. Biomaterials.

[43] Wu J, Chen J, Feng Y (2020). An immune cocktail therapy to realize multiple boosting of the cancer-immunity cycle by combination of drug/gene delivery nanoparticles. Sci Adv.

[44] Yamazaki T, Pitt JM, Vétizou M (2016). The oncolytic peptide LTX-315 overcomes resistance of cancers to immunotherapy with CTLA4 checkpoint blockade. Cell Death Differ.

[45] Li J, Wang H, Wang Y (2020). Tumor-activated size-enlargeable bioinspired lipoproteins access cancer cells in tumor to elicit anti-tumor immune responses. Adv Mater.

[46] Liu H, Hu Y, Sun Y (2019). Co-delivery of bee venom melittin and a photosensitizer with an organic-inorganic hybrid nanocarrier for photodynamic therapy and immunotherapy. ACS Nano.

[47] Tang B, Yan R, Zhu J (2022). Integrative analysis of the molecular mechanisms, immunological features and immunotherapy response of ferroptosis regulators across 33 cancer types. Int J Biol Sci.

[48] Ouyang S, Li H, Lou L (2022). Inhibition of STAT3-ferroptosis negative regulatory axis suppresses tumor growth and alleviates chemoresistance in gastric cancer. Redox Biol.

[49] Schreiber R, Ousingsawat J, Wanitchakool P (2018). Regulation of TMEM16A/ANO1 and TMEM16F/ANO6 ion currents and phospholipid scrambling by Ca2+ and plasma membrane lipid. J Physiol.

[50] Yin J, Zhan J, Hu Q, Huang S, Lin W (2023). Fluorescent probes for ferroptosis bioimaging: advances, challenges, and prospects. Chem Soc Rev.

[51] Schreiber R, Buchholz B, Kraus A (2019). Lipid peroxidation drives renal cyst growth in vitro through activation of TMEM16A. J Am Soc Nephrol.

[52] Simões F, Ousingsawat J, Wanitchakool P (2018). CFTR supports cell death through ROS-dependent activation of TMEM16F (anoctamin 6). Pflugers Arch.

[53] Li X, Zhu S, Li Z (2022). Melittin induces ferroptosis and ER stress-CHOP-mediated apoptosis in A549 cells. Free Radic Res.

[54] Cole JM, Dahl R, Cowden Dahl KD (2021). MAPK signaling is required for generation of tunneling nanotube-like structures in ovarian cancer cells. Cancers.

[55] Han IH, Jeong C, Yang J, Park SH, Hwang DS, Bae H (2022). Therapeutic effect of melittin-dKLA targeting tumor-associated macrophages in melanoma. Int J Mol Sci.

[56] Yu X, Chen L, Liu J (2019). Immune modulation of liver sinusoidal endothelial cells by melittin nanoparticles suppresses liver metastasis. Nat Commun.

[57] Yu X, Dai Y, Zhao Y (2020). Melittin-lipid nanoparticles target to lymph nodes and elicit a systemic anti-tumor immune response. Nat Commun.

[58] Gajski G, Domijan AM, Žegura B (2016). Melittin induced cytogenetic damage, oxidative stress and changes in gene expression in human peripheral blood lymphocytes. Toxicon.

[59] Tiwari R, Tiwari G, Lahiri A, Ramachandran V, Rai A (2022). Melittin: a natural peptide with expanded therapeutic applications. Nat Prod J.

[60] Lee MT, Sun TL, Hung WC, Huang HW (2013). Process of inducing pores in membranes by melittin. Proc Natl Acad Sci U S A.

[61] Chiou PC, Hsu WW, Chang Y, Chen YF (2022). Molecular packing of lipid membranes and action mechanisms of membrane-active peptides. Colloids Surf B Biointerfaces.

[62] Clifton LA, Campbell RA, Sebastiani F (2020). Design and use of model membranes to study biomolecular interactions using complementary surface-sensitive techniques. Adv Colloid Interface Sci.

[63] Tuerkova A, Kabelka I, Králová T (2020). Effect of helical kink in antimicrobial peptides on membrane pore formation. Elife.

[64] Maher S, McClean S (2006). Investigation of the cytotoxicity of eukaryotic and prokaryotic antimicrobial peptides in intestinal epithelial cells in vitro. Biochem Pharmacol.

[65] Černe K, Erman A, Veranič P (2013). Analysis of cytotoxicity of melittin on adherent culture of human endothelial cells reveals advantage of fluorescence microscopy over flow cytometry and haemocytometer assay. Protoplasma.

[66] Ownby CL, Powell JR, Jiang MS, Fletcher JE (1997). Melittin and phospholipase A2 from bee (Apis mellifera) venom cause necrosis of murine skeletal muscle in vivo. Toxicon.

[67] Saeed W, Khalil E (2017). Toxic effects and safety of bee venom protein[melittin] in mice: search for natural vaccine adjuvants. J Nat Prod Resour.

[68] Jia F, Chen P, Wang D (2021). bottlebrush polymer-conjugated melittin exhibits enhanced antitumor activity and better safety profile. ACS Appl Mater Interfaces.

[69] Lv Y, Chen X, Chen Z (2022). Melittin tryptophan substitution with a fluorescent amino acid reveals the structural basis of selective antitumor effect and subcellular localization in tumor cells. Toxins.

[70] Oddo A, Hansen PR (2017). Hemolytic activity of antimicrobial peptides. Methods Mol Biol.

[71] Blondelle SE, Simpkins LR, Pérez-Payá E, Houghten RA (1993). Influence of tryptophan residues on melittin’s hemolytic activity. Biochim Biophys Acta.

[72] Asthana N, Yadav SP, Ghosh JK (2004). Dissection of antibacterial and toxic activity of melittin: a leucine zipper motif plays a crucial role in determining its hemolytic activity but not antibacterial activity. J Biol Chem.

[73] Periayah MH, Halim AS, Mat Saad AZ (2017). Mechanism action of platelets and crucial blood coagulation pathways in hemostasis. Int J Hematol Oncol Stem Cell Res.

[74] Zolfagharian H, Mohajeri M, Babaie M (2015). Honey bee venom (Apis mellifera) contains anticoagulation factors and increases the blood-clotting time. J Pharmacopuncture.

[75] Mingomataj EC, Bakiri AH (2012). Episodic hemorrhage during honeybee venom anaphylaxis: potential mechanisms. J Investig Allergol Clin Immunol.

[76] Kim Y, Lee YW, Kim H, Chung DK (2019). Bee venom alleviates atopic dermatitis symptoms through the upregulation of decay-accelerating factor (DAF/CD55). Toxins.

[77] Drag M, Salvesen GS (2010). Emerging principles in protease-based drug discovery. Nat Rev Drug Discov.

[78] Yaacoub C, Wehbe R, Salma Y (2022). Apis mellifera syriaca Venom: evaluation of Its anticoagulant effect, proteolytic activity, and cytotoxicity along with its two main compounds-MEL and PLA2-On HeLa cancer cells. Molecules.

[79] Lee J, Park J, Yeom J, Han EH, Lim YH (2017). Inhibitory effect of bee venom on blood coagulation via anti-serine protease activity. J Asia Pac Entomol.

[80] Wang Y, Zhai W, Cheng S (2023). Surface-functionalized design of blood-contacting biomaterials for preventing coagulation and promoting hemostasis. Friction.

[81] Prado M, Solano Trejos G, Lomonte B (2010). Acute physiopathological effects of honeybee (Apis mellifera) envenoming by subcutaneous route in a mouse model. Toxicon.

[82] Kołaczek A, Skorupa D, Antczak-Marczak M, Kuna P, Kupczyk M (2017). Safety and efficacy of venom immunotherapy: a real life study. Postepy Dermatol Alergol.

[83] Lee G, Bae H (2016). Anti-inflammatory applications of melittin, a major component of bee venom: detailed mechanism of action and adverse effects. Molecules.

[84] Hartmann A, Müllner J, Meier N (2016). Bee venom for the treatment of parkinson disease—a randomized controlled clinical trial. PLoS ONE.

[85] Antolín-Amérigo D, Ruiz-León B, Boni E (2018). Component-resolved diagnosis in hymenoptera allergy. Allergol Immunopathol.

[86] Ding J, Xiao Y, Lu D, YR DU, Cui XY, Chen J (2011). Effects of SKF-96365, a TRPC inhibitor, on melittin-induced inward current and intracellular Ca2+ rise in primary sensory cells. Neurosci Bull.

[87] Lu ZM, Xie F, Fu H (2008). Roles of peripheral P2X and P2Y receptors in the development of melittin-induced nociception and hypersensitivity. Neurochem Res.

[88] Ding J, Zhang JR, Wang Y (2012). Effects of a non-selective TRPC channel blocker, SKF-96365, on melittin-induced spontaneous persistent nociception and inflammatory pain hypersensitivity. Neurosci Bull.

[89] Jung H, Kim YS, Jung DM, Lee KS, Lee JM, Kim KK (2022). Melittin-derived peptides exhibit variations in cytotoxicity and antioxidant, anti-inflammatory and allergenic activities. Anim Cells Syst.

[90] Perekalin DS, Novikov VV, Pavlov AA (2015). Selective ruthenium labeling of the tryptophan residue in the bee venom peptide melittin. Chemistry.

[91] Grześkowiak BF, Maziukiewicz D, Kozłowska A, Kertmen A, Coy E, Mrówczyński R (2021). Polyamidoamine dendrimers decorated multifunctional polydopamine nanoparticles for targeted chemo- and photothermal therapy of liver cancer model. Int J Mol Sci.

[92] Lu D, Wang J, Li Y (2020). Tumor noninvasive and target embolization therapy platform by intravenous injection based on acidic microenvironment-responsive hyperbranched poly(amino acid)s. ACS Cent Sci.

[93] Yu G, Zhang M, Saha ML (2017). Antitumor activity of a unique polymer that incorporates a fluorescent self-assembled metallacycle. J Am Chem Soc.

[94] Yang M, Li J, Gu P, Fan X (2020). The application of nanoparticles in cancer immunotherapy: targeting tumor microenvironment. Bioact Mater.

[95] Gentile EA, Castronuovo CC, Cuestas ML (2019). F127 poloxamer effect on cytotoxicity induction of tumour cell cultures treated with doxorubicin. J Pharm Pharmacol.

[96] Movassaghian S, Merkel OM, Torchilin VP (2015). Applications of polymer micelles for imaging and drug delivery. Wiley Interdiscip Rev Nanomed Nanobiotechnol.

[97] Xu Y, Deng M, Zhang H (2020). Selection of affinity reagents to neutralize the hemolytic toxicity of melittin based on a self-assembled nanoparticle library. ACS Appl Mater Interfaces.

[98] Sharma G, Valenta DT, Altman Y (2010). Polymer particle shape independently influences binding and internalization by macrophages. J Control Release.

[99] Wang H, Wang S, Wang R (2019). Co-delivery of paclitaxel and melittin by glycopeptide-modified lipodisks for synergistic anti-glioma therapy. Nanoscale.

[100] Belluati A, Mikhalevich V, Yorulmaz Avsar S (2020). How do the properties of amphiphilic polymer membranes influence the functional insertion of peptide pores?. Biomacromol.

[101] Cui Y, Sun J, Hao W (2020). Dual-target peptide-modified erythrocyte membrane-enveloped PLGA nanoparticles for the treatment of glioma. Front Oncol.

[102] Thangudu S, Yu CC, Lee CL, Liao MC, Su CH (2022). Magnetic, biocompatible FeCO3 nanoparticles for T2-weighted magnetic resonance imaging of in vivo lung tumors. J Nanobiotechnol.

[103] Li H, Wang D, Zhou X (2022). Characterization of spleen and lymph node cell types via CITE-seq and machine learning methods. Front Mol Neurosci.

[104] Hu H, Wang J, Wang H (2018). Cell-penetrating peptide-based nanovehicles potentiate lymph metastasis targeting and deep penetration for anti-metastasis therapy. Theranostics.

[105] Lan HR, Zhang YN, Han YJ (2023). Multifunctional nanocarriers for targeted drug delivery and diagnostic applications of lymph nodes metastasis: a review of recent trends and future perspectives. J Nanobiotechnol.

[106] Cabral H, Makino J, Matsumoto Y (2015). Systemic targeting of lymph node metastasis through the blood vascular system by using size-controlled nanocarriers. ACS Nano.

[107] Huang C, Jin H, Qian Y (2013). Hybrid melittin cytolytic peptide-driven ultrasmall lipid nanoparticles block melanoma growth in vivo. ACS Nano.

[108] Rahimi S, Chen Y, Zareian M, Pandit S, Mijakovic I (2022). Cellular and subcellular interactions of graphene-based materials with cancerous and non-cancerous cells. Adv Drug Deliv Rev.

[109] Qiao H, Mei J, Yuan K (2022). Immune-regulating strategy against rheumatoid arthritis by inducing tolerogenic dendritic cells with modified zinc peroxide nanoparticles. J Nanobiotechnology.

[110] Daniluk K, Lange A, Wójcik B (2023). Effect of melittin complexes with graphene and graphene oxide on triple-negative breast cancer tumors grown on chicken embryo chorioallantoic membrane. Int J Mol Sci.

[111] Shin MC, Min KA, Cheong H (2016). Preparation and characterization of gelonin-melittin fusion biotoxin for synergistically enhanced anti-tumor activity. Pharm Res.

[112] Wang D, Hu L, Su M, Wang J, Xu T (2015). Preparation and functional characterization of human vascular endothelial growth factor-melittin fusion protein with analysis of the antitumor activity in vitro and in vivo. Int J Oncol.

[113] Sun D, Sun M, Zhu W, Wang Z, Li Y, Ma J (2015). The anti-cancer potency and mechanism of a novel tumor-activated fused toxin. DLM Toxins.

[114] Jeong C, Kim J, Han IH (2023). Melittin derived peptide-drug conjugate, M-DM1, inhibits tumor progression and induces effector cell infiltration in melanoma by targeting M2 tumor-associated macrophages. Front Immunol.

[115] Duffy C, Sorolla A, Wang E (2020). Honeybee venom and melittin suppress growth factor receptor activation in HER2-enriched and triple-negative breast cancer. NPJ Precis Oncol.

[116] Lee C, Bae SS, Joo H, Bae H (2017). Melittin suppresses tumor progression by regulating tumor-associated macrophages in a Lewis lung carcinoma mouse model. Oncotarget.

[117] Lee C, Jeong H, Bae Y (2019). Targeting of M2-like tumor-associated macrophages with a melittin-based pro-apoptotic peptide. J Immunother Cancer.

[118] Maani Z, Farajnia S, Rahbarnia L, Hosseingholi EZ, Khajehnasiri N, Mansouri P (2023). Rational design of an anti-cancer peptide inhibiting CD147 / Cyp A interaction. J Mol Struct.

[119] Rajabnejad SH, Mokhtarzadeh A, Abnous K, Taghdisi SM, Ramezani M, Razavi BM (2018). Targeted delivery of melittin to cancer cells by AS1411 anti-nucleolin aptamer. Drug Dev Ind Pharm.

[120] Yazdian-Robati R, Arab A, Ramezani M (2019). Smart aptamer-modified calcium carbonate nanoparticles for controlled release and targeted delivery of epirubicin and melittin into cancer cells in vitro and in vivo. Drug Dev Ind Pharm.

[121] Bahreyni A, Mohamud Y, Zhang J, Luo H (2023). Engineering a facile and versatile nanoplatform to facilitate the delivery of multiple agents for targeted breast cancer chemo-immunotherapy. Biomed Pharmacother.

[122] Dai Y, Yu X, Leng Y (2023). Effective treatment of metastatic sentinel lymph nodes by dual-targeting melittin nanoparticles. J Nanobiotechnology.

[123] Curcio M, Vittorio O, Bell JL, Iemma F, Nicoletta FP, Cirillo G (2022). hyaluronic acid within self-assembling nanoparticles: endless possibilities for targeted cancer therapy. Nanomaterials.

[124] Jia HR, Zhu YX, Xu KF, Wu FG (2018). Turning toxicants into safe therapeutic drugs: cytolytic peptide-photosensitizer assemblies for optimized in vivo delivery of melittin. Adv Healthc Mater.

[125] Motiei M, Aboutalebi F, Forouzanfar M, Dormiani K, Nasr-Esfahani MH, Mirahmadi-Zare SZ (2021). Smart co-delivery of miR-34a and cytotoxic peptides (LTX-315 and melittin) by chitosan based polyelectrolyte nanocarriers for specific cancer cell death induction. Mater Sci Eng C Mater Biol Appl.

[126] Gao J, Wang S, Dong X, Wang Z (2021). RGD-expressed bacterial membrane-derived nanovesicles enhance cancer therapy via multiple tumorous targeting. Theranostics.

[127] Xiao H, Zhang R, Fan X (2022). Super-sensitive bifunctional nanoprobe: Self-assembly of peptide-driven nanoparticles demonstrating tumor fluorescence imaging and therapy. Acta Pharm Sin B.

[128] Ye R, Zheng Y, Chen Y (2021). Stable loading and delivery of melittin with lipid-coated polymeric nanoparticles for effective tumor therapy with negligible systemic toxicity. ACS Appl Mater Interfaces.

[129] Wang T, Wu C, Hu Y, Zhang Y, Ma J (2023). Stimuli-responsive nanocarrier delivery systems for Pt-based antitumor complexes: a review. RSC Adv.

[130] Ashrafizadeh M, Delfi M, Zarrabi A (2022). Stimuli-responsive liposomal nanoformulations in cancer therapy: pre-clinical & clinical approaches. J Control Release.

[131] Shahriari M, Zahiri M, Abnous K, Taghdisi SM, Ramezani M, Alibolandi M (2019). Enzyme responsive drug delivery systems in cancer treatment. J Control Release.

[132] Cao H, Wang H, He X (2018). Bioengineered macrophages can responsively transform into nanovesicles to target lung metastasis. Nano Lett.

[133] Maher S, Feighery L, Brayden DJ, McClean S (2007). Melittin as a permeability enhancer II: in vitro investigations in human mucus secreting intestinal monolayers and rat colonic mucosae. Pharm Res.

[134] Maher S, Devocelle M, Ryan S, McClean S, Brayden DJ (2010). Impact of amino acid replacements on in vitro permeation enhancement and cytotoxicity of the intestinal absorption promoter, melittin. Int J Pharm.

[135] Wang J, Wang Y, Cao H (2020). Orally delivered legumain-activated nanovehicles improve tumor accumulation and penetration for combinational photothermal-chemotherapy. J Control Release.

[136] Liu X, Song H, Sun T, Wang H (2023). Responsive microneedles as a new platform for precision immunotherapy. Pharmaceutics.

[137] Sun Q, Chen W, Wang M (2023). A chase and block strategy for enhanced cancer therapy with hypoxia-promoted photodynamic therapy and autophagy inhibition based on upconversion nanocomposites. Adv Healthc Mater.

[138] Zhang J, Liu X, Xia Y (2023). Genetically engineered nano-melittin vesicles for multimodal synergetic cancer therapy. Bioeng Transl Med.

[139] Ding C, Chen C, Zeng X, Chen H, Zhao Y (2022). Emerging strategies in stimuli-responsive prodrug nanosystems for cancer therapy. ACS Nano.

[140] Cheng B, Xu P (2020). Redox-sensitive nanocomplex for targeted delivery of melittin. Toxins.

[141] Lee CM, Lee J, Nam MJ, Choi YS, Park SH (2019). Tomentosin displays anti-carcinogenic effect in human osteosarcoma mg-63 cells via the induction of intracellular reactive oxygen species. Int J Mol Sci.

[142] Li J, Zhao J, Tan T (2020). Nanoparticle drug delivery system for glioma and its efficacy improvement strategies: a comprehensive review. Int J Nanomedicine.

[143] Hubert P, Roncarati P, Demoulin S (2021). Extracellular HMGB1 blockade inhibits tumor growth through profoundly remodeling immune microenvironment and enhances checkpoint inhibitor-based immunotherapy. J Immunother Cancer.

[144] Qiao H, Fang D, Zhang L (2018). Nanostructured peptidotoxins as natural Pro-oxidants induced cancer cell death via amplification of oxidative stress. ACS Appl Mater Interfaces.

[145] Luo L, Wu W, Sun D (2018). Acid-activated melittin for targeted and safe antitumor therapy. Bioconjug Chem.

[146] Song K, Nguyen DC, Luu T (2023). A mannosylated polymer with endosomal release properties for peptide antigen delivery. J Control Release.

[147] Seynhaeve ALB, Amin M, Haemmerich D, van Rhoon GC, Ten Hagen TLM (2020). Hyperthermia and smart drug delivery systems for solid tumor therapy. Adv Drug Deliv Rev.

[148] Koide H, Saito K, Yoshimatsu K (2023). Cooling-induced, localized release of cytotoxic peptides from engineered polymer nanoparticles in living mice for cancer therapy. J Control Release.

[149] Maitip J, Mookhploy W, Khorndork S, Chantawannakul P (2021). Comparative study of antimicrobial properties of bee venom extracts and melittins of honey bees. Antibiotics.

[150] Liu Z, Fan Z, Liu J (2023). Melittin-carrying nanoparticle suppress T cell-driven immunity in a murine allergic dermatitis model. Adv Sci.

[151] Sylvestre M, Lv S, Yang LF (2021). Replacement of L-amino acid peptides with D-amino acid peptides mitigates anti-PEG antibody generation against polymer-peptide conjugates in mice. J Control Release.

